# Precise characterization of somatic complex structural variations from tumor/control paired long-read sequencing data with nanomonsv

**DOI:** 10.1093/nar/gkad526

**Published:** 2023-06-20

**Authors:** Yuichi Shiraishi, Junji Koya, Kenichi Chiba, Ai Okada, Yasuhito Arai, Yuki Saito, Tatsuhiro Shibata, Keisuke Kataoka

**Affiliations:** Division of Genome Analysis Platform Development, National Cancer Center Research Institute, Tokyo, Japan; Division of Molecular Oncology, National Cancer Center Research Institute, Tokyo, Japan; Division of Genome Analysis Platform Development, National Cancer Center Research Institute, Tokyo, Japan; Division of Genome Analysis Platform Development, National Cancer Center Research Institute, Tokyo, Japan; Division of Cancer Genomics, National Cancer Center Research Institute, Tokyo, Japan; Division of Molecular Oncology, National Cancer Center Research Institute, Tokyo, Japan; Department of Gastroenterology, Keio University School of Medicine, Tokyo, Japan; Division of Cancer Genomics, National Cancer Center Research Institute, Tokyo, Japan; Laboratory of Molecular Medicine, The Institute of Medical Science, The University of Tokyo, Tokyo, Japan; Division of Molecular Oncology, National Cancer Center Research Institute, Tokyo, Japan; Department of Hematology, Keio University School of Medicine, Tokyo, Japan

## Abstract

We present our novel software, nanomonsv, for detecting somatic structural variations (SVs) using tumor and matched control long-read sequencing data with a single-base resolution. The current version of nanomonsv includes two detection modules, Canonical SV module, and Single breakend SV module. Using tumor/control paired long-read sequencing data from three cancer and their matched lymphoblastoid lines, we demonstrate that Canonical SV module can identify somatic SVs that can be captured by short-read technologies with higher precision and recall than existing methods. In addition, we have developed a workflow to classify mobile element insertions while elucidating their in-depth properties, such as 5′ truncations, internal inversions, as well as source sites for 3′ transductions. Furthermore, Single breakend SV module enables the detection of complex SVs that can only be identified by long-reads, such as SVs involving highly-repetitive centromeric sequences, and LINE1- and virus-mediated rearrangements. In summary, our approaches applied to cancer long-read sequencing data can reveal various features of somatic SVs and will lead to a better understanding of mutational processes and functional consequences of somatic SVs.

## Graphical Abstract

**Graphical Abstract:**
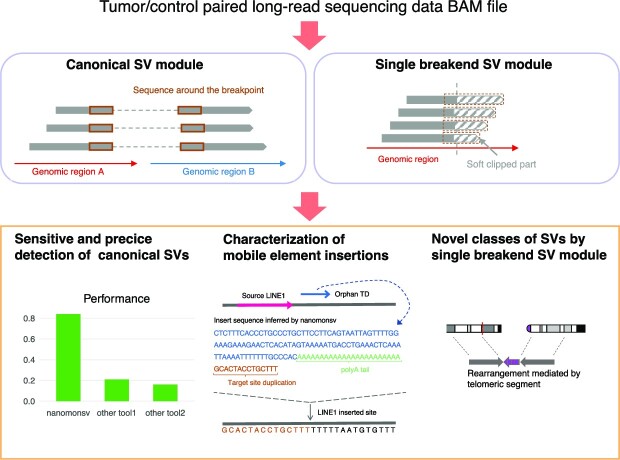

## INTRODUCTION

Structural variations (SVs) have been known to play an important role in cancer pathogenesis. Advances in high-throughput sequencing technologies have enabled us to perform genome-wide somatic SV detection, and a number of cancer-driving SVs have been identified (1–3). On the other hand, millions of repetitive elements are widely distributed throughout the human genome, which hinders unambiguous alignment by current standard short-read technologies. According to several computational predictions, such repeat sequences comprise one-half to two-thirds of the human genome (4,5). Since the majority of the current sequencing data is collected using short-read sequencing technologies, several classes of SVs, especially those whose breakpoints are located in these repeat regions, have been difficult to detect (6,7). As such, although a large number of whole-genome sequencing studies have aimed to detect somatic SVs, it is plausible to assume that the landscape of SVs remains elusive in human cancer.

Recently, long-read sequencing technologies attracted lots of attention with the hope of improving the performance of SV detection (8,9). Several studies have developed SV detection tools and shown the effectiveness of long-read data (10–15). However, most previous studies focused on germline SVs. For identifying somatic SVs, one typical approach is to perform existing algorithms for both tumor and control sequencing data individually and take the subtraction of the set of SVs found in the tumor from that in the matched control. However, this approach can generate many false positives, such as germline SVs that pass the threshold in the tumor and narrowly miss it in the matched control (e.g. because of low sequencing depths). Therefore, algorithms that can detect SVs by jointly utilizing tumor and matched control long-read sequencing data are needed (16,17).

Another important issue that long-read technologies can address is the characterization of the detailed structure of long insertions, especially mobile element insertions (MEIs) including LINE1 retrotransposition (18,19). Among the millions of LINE1 elements existing across the human genome, approximately one hundred are thought to be still active. They can somatically produce their RNA intermediates, which are inserted into distant genomic sites with some modifications (such as 5′ truncations, internal inversions, and 3′ transductions). Besides, LINE1 elements also facilitate the somatic displacement of other mobile elements such as Alu, SINE/VNTR/Alu (SVA), and processed pseudogenes. Short-read sequencing can, in principle, detect the existence of such insertion events, and several studies successfully characterized their roles in cancer (20,21). However, as the range of genomic sequences which can be analyzed by short-read sequencing is limited to a few hundred nucleotides from the edge of inserted sequences, the entire landscape and genetic properties of MEI events have not been fully elucidated.

In this paper, we introduce our approach, nanomonsv (https://github.com/friend1ws/nanomonsv), that can identify somatic SVs with single-nucleotide resolution jointly using both tumor and control long-read sequencing data with Oxford Nanopore Technologies (ONT) and PacBio platform. With this software, we evaluated the effectiveness of long-read sequencing for somatic SV detection using newly collected long-read sequencing data from three pairs of cancer and matched control cell-lines. The characteristics of nanomonsv are summarized as follows:

- Canonical SV module can capture not only most of the SVs that can be identified using short-read sequencing platforms but also additional ones.
- For insertions, the full-length inserted sequences obtained by the nanomonsv allowed us to characterize their genetic properties (such as 5′ truncations, internal inversions, and target site duplications) and to identify source sites for 3′ transduction mediated by LINE1.
- Single breakend SV module of nanomonsv can identify single breakend SVs where only one breakpoint is identified because the other breakpoint is typically located in repetitive regions. Examples of single breakend SVs include LINE1-mediated rearrangements, rearrangements associated with centromeric regions, and viral integrations.

## MATERIALS AND METHODS

### Whole genome sequencing using oxford nanopore technologies and illumina novaseq 6000

The cell-lines used in this study (COLO829, COLO829BL, H2009, BL2009, HCC1954 and HCC1954BL) were obtained from ATCC (American Type Culture Collection). For Oxford Nanopore Technologies (ONT) sequencing data, high-molecular-weight (HMW) genomic DNAs were extracted from these cell-lines with QIAGEN Genomic-tip 500/G (QIAGEN). HBV-positive liver cancer cell-line PRC/PRF/5 was obtained from the JCRB cell bank (National Institutes of Biomedical Innovation, Health and Nutrition), and HMW-genomic DNA was isolated using SmartDNA chip (Analytik Jena). DNA libraries were then prepared using the Ligation Sequencing Kit 1D and sequenced on the PromethION platform with R9.4.1 flow cells, to generate fast5 files. Then, these fast5 files were base-called and converted to FASTQ files using Guppy 3.4.5. Then, these were aligned by minimap2 with ‘-ax map-ont -t 8 -p 0.1' option to the human reference genome provided at the Genomic Data Commons website (GRCh38.d1.vd1). Summary statistics were calculated using NanoStat package (22) after removing secondary and supplementary alignments from BAM files.

For Illumina short-read sequencing data, we performed Illumina Novaseq 6000 with a standard 150 bp paired-end read protocol, and these were aligned by BWA-MEM (23) version 0.1.17 to the same human reference genome and were sorted by the genomic coordinates, followed by removal of PCR duplicates via biobambam (https://github.com/gt1/biobambam) version 0.0.191 as previously described (24). In addition, we performed somatic structural variation detection using manta (25), SvABA (26), GRIDSS (27,28), GenomonSV, and TraFiC-mem (20) (see Supplementary Text for detail).

### Overview of nanomonsv

Here we describe an overview of nanomonsv. A more detailed description of the algorithm can be found in the Supplementary Text. In this paper, SVs were largely classified into two categories:

- Canonical SV: SVs characterized by two breakpoints and inserted sequences between them. These SVs include insertions where two breakpoints are typically close together.
- Single breakend SV: SVs characterized by a single breakpoint and the sequence after the breakpoint, which are often not uniquely aligned to the reference genome, and their positions are not precisely located.

**Figure 1.**
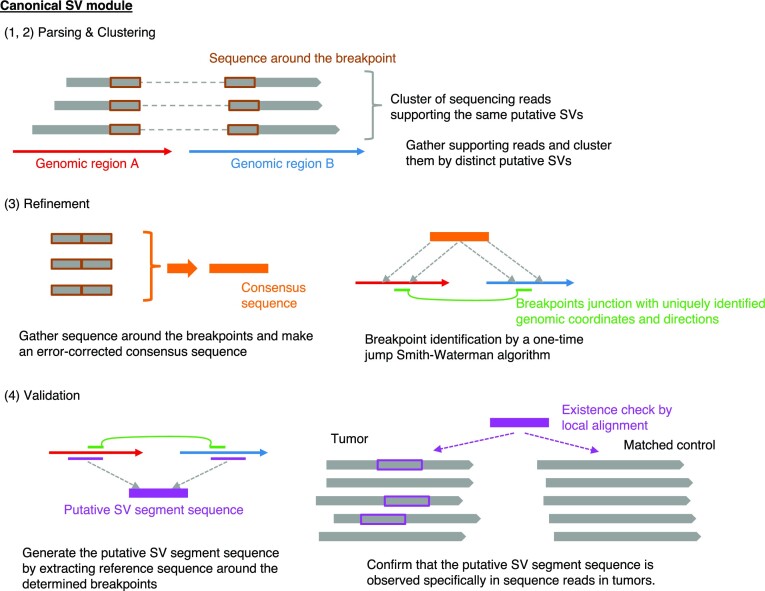
Workflow of somatic SV detection in nanomonsv Canonical SV module. Canonical SV module for nanomonsv consists of the following four steps. Parsing: the reads likely supporting SVs are extracted from both tumor and matched control BAM files using CIGAR string and supplementary alignment information. Clustering: the reads from the tumor sample that presumably span the same SVs are clustered, and the possible ranges of breakpoints are inferred for each possible SV. If there exist apparent supporting reads in the matched control sample (or non-matched control panel samples when they are available), these are also removed. Refinement: Extract the portions of the supporting reads around the breakpoints, and perform error-correction using racon (78) to generate a consensus sequence for each candidate SV. Then, aligning the consensus sequence to those around the possible breakpoint regions in the reference genome using a modified Smith-Waterman algorithm (which allows a one-time jump from one genomic region to the other, see Supplementary Figure S1), we identify the exact breakpoint positions and the inserted sequence inside them. Validation: From the breakpoint determined in the previous step, we generate the ‘putative SV segment sequence.’ Then we collect the reads around the breakpoint of putative SVs and check whether the putative SV segment sequence exists (then the read is set as a ‘variant supporting read’) or not (then the read is classified to a ‘reference read’) in each read of the tumor and matched control. Finally, candidate SVs with ≥3 variants supporting reads in the tumor and no variant supporting reads in the matched control sample are kept as the final SVs. See Supplementary Text for detail.

**Figure 2.**
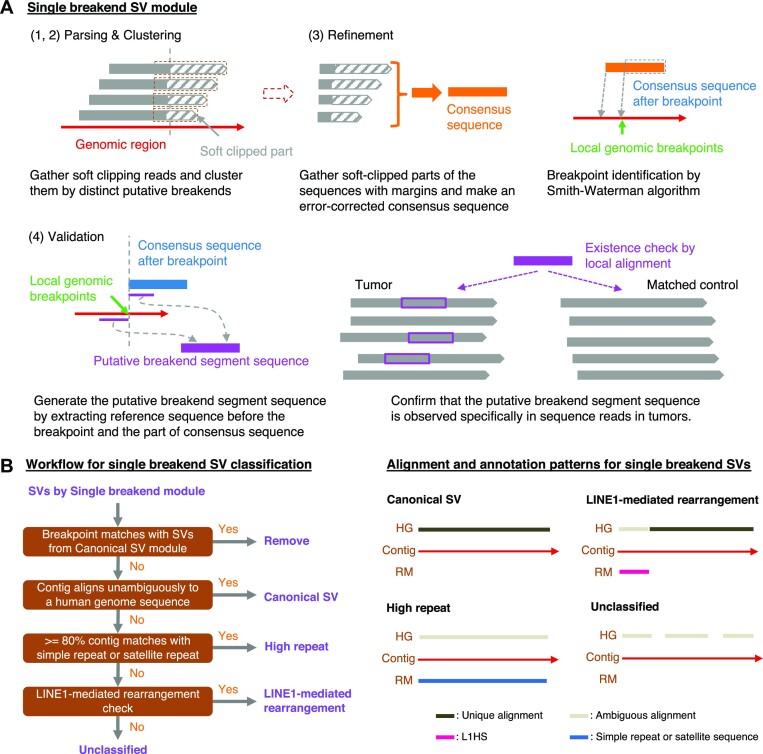
Workflow of somatic SV detection and classification in nanomonsv single breakend SV module. (**A**) Single breakend SV module for nanomonsv consists of the following four steps. Parsing: the reads putatively supporting single breakend SVs are extracted from both tumor and matched control BAM files using soft clipping information in the CIGAR strings. Clustering: the reads from the tumor sample that presumably support the same single breakend SVs are clustered. The candidates are removed if apparent supporting reads are detected in the matched control sample (or non-matched control panel samples when they are available). Refinement: Gather the soft-clipped part of the reads with 100 bp margins inside the breakpoints and generate an error-corrected consensus sequence by two round iterations of all-vs-all alignment by minimap2 (29) and polishing with racon (78). Then, aligning the consensus sequence to those around the possible breakpoint regions by Smith-Waterman algorithm, we detect single base resolution breakpoints and the consensus sequence after the breakpoint. Validation: from the breakpoint determined in the previous step and the error-corrected consensus sequence after the breakpoint, we generate the ‘putative SV segment sequence.’ Then, as with Canonical SV module, the reads around the breakpoint of putative single breakend SVs are classified into ‘variant supporting read’ or ‘reference read’ for both tumor and matched control. Finally, candidate SVs with ≥3 variants supporting reads in the tumor and no variant supporting reads in the matched control sample are kept as the final single breakend SVs. See Supplementary Text for detail. (**B**) The left panel shows the chart for classifying SVs identified by Single breakend module. After removing SVs that share a breakpoint with SVs already detected via Canonical SV module, SVs are basically classified by integrating the alignment of contig sequences to the human reference genome (HG) and the annotation results by RepeatMasker (RM). The right panel shows the typical pattern of an alignment to HG and an annotation result by RM of the contig for each category. L1HS stands for the human LINE-1 (L1) element L1 Homo sapiens (L1Hs).

Nanomonsv consists of two related detection modules designed to detect each of the above SVs; Canonical SV module and Single breakend SV module. Prior to performing nanomonsv, we assume that both tumor and control sequence files are already aligned to the reference genomes with minimap2 (29). The procedures of Canonical SV module and Single breakend SV module are depicted in Figures 1 and 2A, respectively.

Both modules consist of four steps: parsing, clustering, refinement, and validation. In the ‘clustering’ step, the reads from the tumor sample that presumably cover the same SVs are clustered as SV candidates with possible breakpoint ranges. If we observe apparent supporting reads in the matched control sample or in non-matched control panel samples (30 Nanopore sequencing data from the Human Pangenome Reference Consortium (30) are used in this study), these are eliminated. The ‘refinement’ step in Canonical SV module plays an essential role in determining the single-nucleotide resolution breakpoints as well as error-corrected inserted sequences using the modified Smith-Waterman algorithm, which allows one-time jump from one genomic region to the other (see Supplementary Figure S1 and similar algorithm in a previous study (31)). Particularly, polished inserted sequences are beneficial for classifying and characterizing insertion events. The last ‘validation’ steps in both modules thoroughly confirm whether the candidate SV is truly specific for the tumor. More specifically, aligning the putative SV segment sequence to each read close to putative breakpoints enables precise detection of variant supporting reads, especially those partially covering the breakpoints and not counted in the parsing step (similar approaches have been attempted in previous studies (32), albeit in a different context than somatic SV confirmation). Lastly, for deletions and insertions, we focus on those whose sizes are 100 bp or larger. We also removed deletions and insertions confined within simple repeat regions.

We also developed a workflow to characterize putative single breakend SVs by realigning the consensus sequence to the reference genome and execution of RepeatMasker (Figure 2B, see Supplementary Text for detail). For SVs specifically identified by Single breakend SV module, if their breakpoints on the other side were unambiguously identified, they were reclassified as canonical SVs. They included SVs that were filtered out in Canonical SV module because they did not marginally exceed the threshold in the various filtering steps.

### PCR validation

To generate primer sequences for PCR validation for each canonical somatic SV, we first prepared the sequence template by concatenating 800 bp nucleotides from the first breakpoint, the inserted sequence, and 800 bp nucleotides from the second breakpoint. Then, the Python bindings of Primer3 (33) are performed, setting the sequence target as 25 bp nucleotides from the first breakpoint, the inserted sequence, and 25 bp nucleotides from the second breakpoint. Here, we created five pairs of primer sequences for each primer product size range of 201–300, 301–400, 401–500, …, and 1501–1600. Next, we performed GenomeTester (34) to remove pairs of primer sequences that have too many binding sites (>5 for left or right primers) and too many alternative PCR products (more than two for insertion and deletion and more than one for other types of SVs). Finally, for each somatic SV for validation, we selected one primer pair that has a smaller product size, less number of primer binding sites, and alternative PCR products.

To design a primer for highly repetitive sequences such as centromere and telomere, we selected primer sequences that were expected to occur once in the sample genomes. For example, for these primer sequences, we should be able to observe them about 15 times in a 30x coverage FASTQ sequence (in the haploid reference genome). Therefore, we designed the primer sequence as follows:

- We parsed *k*-mers (*k* = 19) from the original FASTQ, and calculated the histogram for each *k*-mer.
- For each *k*-mer subsequence in the assembled contigs for single breakend SVs, we masked it with ‘N’ if it occurred less than 8 times or more than 50 times.
- We concatenated the pre-breakpoint sequence and assembled contigs (we limited to 2000 bp) masked by the above, and designed primers using primer3 on this sequence.

All PCR reactions were performed in a total of 20 ul volume using 10 ul of Go Taq Master Mix (Promega), 1 ul of each primer (final 0.5 nM), 1 ml of gDNA (20 ng), and 8 ul of double-distilled water. The PCR samples were denatured at 95°C for 2 min, subjected to 40 cycles of amplification (95°C for 30 s, 55°C for 30 s and 72°C for (product size (bp)/1000) min and followed by a final extension step at 72°C. A list of primers is provided in Supplementary Data 3. PCR products were resolved by agarose gel electrophoresis. Representative PCR products were purified using QIAquick Gel Extraction Kit (Qiagen) according to the manufacturers’ recommended protocols. Finally, the purified samples were subjected to direct capillary sequencing (eurofin). All sequence data were analyzed using ApE (https://jorgensen.biology.utah.edu/wayned/ape/) and the Chromas Lite viewer (Technlysium Pty., Ltd.).

### Evaluation of nanomonsv using benchmark dataset and simulation

For highly reliable somatic SV sets, we used two datasets. The one is high-confidence somatic SV files obtained from the high-coverage NovaSeq data (35) (https://www.nygenome.org/bioinformatics/3-cancer-cell-lines-on-2-sequencers/COLO-829-NovaSeq–COLO-829BL-NovaSeq.sv.annotated.v6.somatic.high_confidence.final.bedpe). The other is from somatic SV truth set generated by multi-platform and experimental validation (36) (truthset_somaticSVs_COLO829.vcf available at https://zenodo.org/record/3988185), which is converted to GRCh38 coordinates with liftOver. We removed insertions and deletions with ≤100 bp lengths because these were the out-of-score in this paper. For high coverage Nanopore sequence data (ERR2752451, ERR2752452) and PacBio sequence data (ERR2808247, ERR2808248) of COLO829 and its matched control, we downloaded FASTQ sequencing data of ENA study accession PRJEB27698 (36), and aligned to the reference genome with minimap2 to the GRCh38 reference genome and sorted and indexed using samtools. Then, nanomonsv was performed on these data as described in the previous section.

For the comparison with nanomonsv, we adopted ‘separate detection and subtraction approach’, where we independently applied standard SV detection tools (Sniffles (10,37) (https://github.com/fritzsedlazeck/Sniffles) version 2.0.7, cuteSV (15) version 2.0.0, Delly (38) version 1.0.3, SVIM (13) version 2.0.0) to both tumor and matched control samples with different thresholds, and eliminated the SVs called in matched controls from those found in tumors. We first aligned the FASTQ files of tumor and matched control using minimap2 with the same setting with nanomonsv. Then, we performed Sniiffles, cuteSV, Delly and SVIM on tumor and matched control BAM files, separately. The option of each software were:

- Sniffles: ‘–minsupport 1 –non-germline’
- cuteSV: ‘–max_cluster_bias_INS 100 –diff_ratio_merging_INS 0.3 –max_cluster_bias_DEL 100 –diff_ratio_merging_DEL 0.3 –min_support = 1’
- Delly: ‘lr’ command
- SVIM: ‘alignment’ command with ‘–skip_genotyping’

For each method, we extracted SVs from tumor samples with ≥3 supporting reads (5 ≥ for high coverage data from PRJEB27698) and removed those whose breakpoints overlapped with any of SVs detected from normal samples allowing for 200 bp margins. We also removed SVs confined within simple repeat regions. We also used CAMPHORsomatic (39) (https://github.com/afujimoto/CAMPHORsomatic) on commit 7ad6bdb for somatic SV detection. We applied our own patch (https://github.com/ncc-ccat-gap/module_box_aokad/blob/master/20221005-CAMPHORsomatic/SB_CH.patch) to CAMPHORsomatic since it could not be executed without that modification. We run CAMPHORsomatic with the default setting.

For simulations, we prepared two haploid human genomes; extracted 22 autosomes and chromosome X from the human reference genome (GRCh38), and injected in-silico germline SVs (2500 duplications, 5000 indels, 100 inversions, 50 inversion-deletion, and 50 inversion-duplications) using the ‘simSV’ command of by SURVIVOR (version 1.0.6, https://github.com/fritzsedlazeck/SURVIVOR) (40). Then, we merged the haploid human genomes to make diploid human genomes with germline SVs to constitute an in-silico matched control genome. Then, we further generate ‘somatic SVs’ (100 duplications, 200 indels, 100 translocations and 100 inversions) on the in-silico matched control genomes to make up an in-silico tumor genome. Since the coordinate system of the simulated somatic SVs is based on the in-silico matched control genome, we converted the coordinate system of the simulated somatic SV list back to the GRCh38. Next, we performed NanoSim (41) (https://github.com/bcgsc/NanoSim, version 2.6.0) on this in-silico tumor and matched control genome to generate Nanopore-like tumor and two matched control (one is literally for matched control data and the other is for mixing with tumor sequencing data) sequencing data. After learning the parameters using Nanopore reads of COLO829BL aligned to chromosome 22 via the read_analysis.py script, we generated simulated Nanopore reads with sufficient depths (∼180Gb yields) via the simulator.py script. These FASTQ files were aligned with minimap2 to generate BAM files. Finally, we sub-sampled Nanopore-like BAM files to generate tumor and matched control BAM data with specified sequencing amounts (10x, 20x, 30x, 40x, and 50x) and the tumor purities (0%, 20%, 40%, 60%, 80% and 100%) and performed nanomonsv as well as Sniffles, cuteSV, Delly, SVIM and CAMPHORsomatic as described above to obtain somatic SV calls from each method.

### Methylation analysis

To quantify the amount of methylation, we used nanopolish version 0.11.1 (https://github.com/jts/nanopolish). First, we performed the ‘nanopolish index’ command from the original fast5 file to generate the index that associates read IDs and their signal-devel data. Then, we executed the ‘nanopolish call-methylation’ command to make the TSV file summarizing the log-likelihood ratio for methylation for each read ID and genomic position. Then, we obtained the methylation frequency at each genomic position using the script provided on the software website. To measure the significance of methylation frequency difference between the tumor and the match control at each LINE1 source element, we first calculated the *P*-value at each locus using Fisher's exact test with the alternative hypothesis of one-sided, and then obtained an asymptotically exact *P*-value using harmonicmeanp package version 3.0 (42).

### Calculation of higher-order repeat match score

First, single breakend SVs that are classified as ‘High Repeat single breakend SVs’ and that are mostly annotated with ‘Satellite/centr’ by RepeatMasker are extracted. Next, we executed the StringDecomposer (43) version 1.1.2 for each contig against the final monomer FASTA files generated by HORmon (44) (cen*_monomers.fa files under the monomersFinal directory, downloaded from https://figshare.com/articles/dataset/HORmon/16755097/1). Then, for each chromosome monomer file result (final_decomposition.tsv), the degree of monomer concordance is calculated. More specifically, we read the result the files one line at a time, and if the pre-/post-relationship of the monomers (curated from cen*_hors.tsv files from HORmonHORs directory, see Supplementary Data 1) is consistent, (<end-pos> - <start-pos>) * <identity> / 100 is added, and the divided by the length of the contig is the HOR match score (see Supplementary Figure S2).

## RESULTS

### Comparison with short-read sequencing data

We used three cancer cell-lines (COLO829, H2009 and HCC1954) and their matched controls (COLO829BL, BL2009 and HCC1954BL) for the evaluation (see Table 1 for the detailed description of these cell-lines). Long-read whole-genome sequencing was conducted using GridION and PromethION. The total outputs were 59.13 to 156.30 Gbps, and the N50 sequence lengths ranged from 14 309 to 24 501 bp (see Table 1, Supplementary Figure S3). To compare with a short-read platform, we also performed high-coverage sequencing of these three paired cell-lines using Illumina Novaseq 6000 platform. The total amounts of yield after polymerase chain reaction (PCR) duplication removal were 205.76 Gbps to 484.26 Gbps.

**Table 1.** Summary statistics of long-read (Nanopore) and short-read (Illumina) data from six cell-lines. COLO829 (from a metastatic cutaneous melanoma patient) and COLO829BL (from a lymphoblastoid line of the same patient) have been often used as a benchmark in many previous studies (35,47,77). Although this cell-line has been known to have hypermutated nature for somatic single nucleotide variants as well as double nucleotide ones, the number of somatic SVs seems to be relatively low. H2009 (from metastatic lung adenocarcinoma) has many long insertions mainly by high LINE1 activity and has been used in studies investigating the mechanism of MEIs (20,21). HCC1954 (from ductal breast carcinoma) and HCC1954BL also have been frequently used as a benchmark (TCGA mutation calling benchmark 4, https://gdc.cancer.gov/) and seem to have a relatively large number of somatic SVs. Although these cell-lines have been used in many studies, there have been few efforts to characterize exhaustive and accurate lists of somatic SVs from these cell-lines

Cell-line	Long-read yield (Gbp)	Long-read total read count	Long-read median read length	Long-read max read length	Long-read N50 length	Short-read yield (Gbp)
COLO829	67.17	5,176,983	7,997	185,650	24,138	250.28
COLO829BL	59.13	6,253,574	5,691	124,349	17,243	393.24
H2009	114.91	10,319,362	6,342	238,152	20,873	484.26
BL2009	156.30	15,684,323	5,195	240,066	20,337	319.82
HCC1954	145.58	11,285,481	7,523	250,253	24,501	291.86
HCC1954BL	126.34	17,608,439	3,689	220,506	14,309	205.76

**Figure 3.**
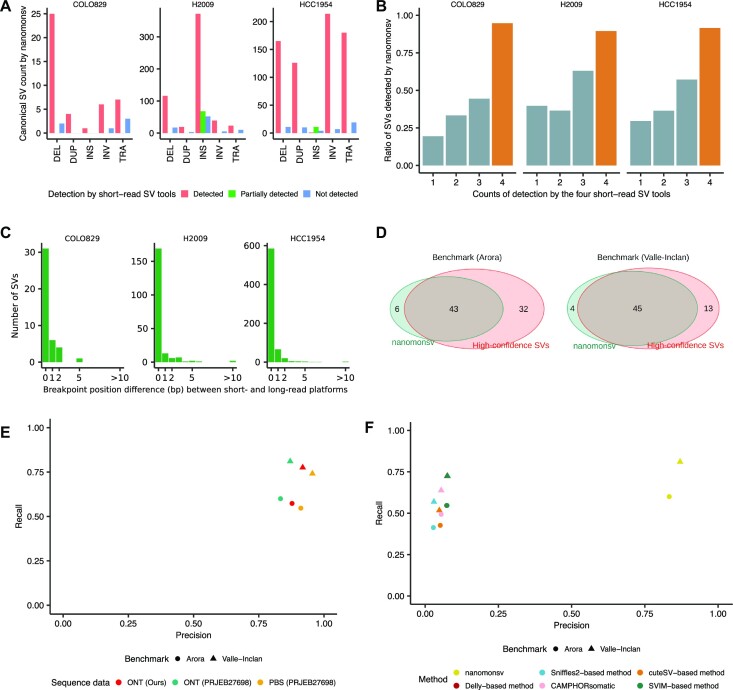
Overview of somatic SVs identified by nanomonsv and their performance evaluations. (**A**) The number of somatic SVs detected by nanomonsv grouped by the type of SVs and whether they are identified by the short-read analysis. DEL, DUP, INS, INV and TRA stand for deletion, duplication, insertion, inversion, and translocation, respectively. Here, ‘partially detected’ indicates the case where either of the two breakpoints is the same as the one detected from short-read. A typical example includes an INS whose inserted sequences came from the other part of the genome, and one of the breakpoints could be identified as a different type of SV (usually as TRA) by short-read. (**B**) The ratio of somatic SVs identified by nanomonsv among those detected by the short-read platform stratified by how often these SVs are called by four software programs (manta, SvABA, GRIDSS, and GenomonSV). Also, the ratio of SVs detected by all four programs (list of highly reliable SVs) was highlighted with a different color (dark orange). (**C**) Histogram of the number of SVs according to the deviations of breakpoint positions from a short-read platform. (**D**) Overlap between SVs detected by nanomonsv and high-confidence SVs in COLO829 determined by two benchmark datasets [SVs detected from high coverage Illumina sequence data (Arora *et al.* 2019) and SVs detected and validated by multiple platforms and experiments (Valle-Inclan et al. 2020)]. (**E**) Precision and recall of nanomonsv measured using two benchmark datasets (Arora *et al.* 2019, Valle-Inclan et al. 2020), assuming that SVs not present in the benchmark are all false positives. Performance was measured using three pairs of COLO829 sequencing data, consisting of our data [ONT (Ours)], high coverage ONT [ONT (PRJEB27698)], and PacBio sequencing data [PBS (PRJEB27698)]. (**F**) Precision and recall measured by four different approaches (nanomonsv, CAMPHORsomatic and four separate detection and subtraction approaches using Sniffles2, cuteSV, Delly, and SVIM) on our COLO829 dataset. The precision and recall were measured by two benchmark datasets.

Applying nanomonsv to these long-read data and rescuing canonical SVs identified from Single breakend SV module, we identified 49, 724 and 748 canonical SVs for COLO829, H2009 and HCC1954, respectively (Figure 3A, Supplementary Figure S4, Supplementary Data 2). Those included 39 SVs that were specifically identified by Single breakend SV module and reclassified into canonical SVs. For the evaluation of precision, we performed the PCR on 139 randomly selected SVs, and 132 (94.9%) showed tumor sample-specific bands with predicted product sizes (see Supplementary Data 3, Supplementary Figures S5, 6). Except for insertions, the validated ratio was reasonably high [96.1% (99/103)]. A relatively low validation ratio for insertions [89.92% (33/37)] might be partly due to the larger size of their PCR products. Even for the insertions not validated by PCR, we observed tumor-specific supporting reads by manual inspection with a genome viewer (45) in most cases (Supplementary Figure S5c). To evaluate recall, we compared with SVs commonly detected by four algorithms (manta (25), SvABA (26), GRIDSS (27,28) and GenomonSV) in the short-read platform, which were considered to be ‘true’ somatic SVs with a high degree of accuracy. Among the total 685 SVs by all four algorithms, nanomonsv applied to ONT sequencing data identified 624 SVs (91.1%) (Figure 3B), suggesting the high sensitivity of nanomonsv on long-read sequencing data even for relatively low coverage compared to short-read sequencing data.

For COLO829, H2009, and HCC1954, 6, 87 and 51 (7.1–12.0%), respectively, were newly detected by long-read sequencing data (not identified by any of the four algorithms or by TraFiC-mem (20) applied to high-coverage Illumina short-read sequencing data). These long-read specific SVs were also validated by PCR method with similar accuracy as SVs detected in the short-read technology (Supplementary Figure S5a). These long-read specific SVs were mostly insertions or SVs with two breakpoints located in repeat or low-complexity regions (Supplementary Figure S7). For instance, the somatic translocation connecting chromosomes 3 and 6 (chr3:26390429–chr6:26193811) in COLO829 was missed by Illumina sequence data, probably because the short-read alignment was highly ambiguous around the breakpoint of chromosome 3 (overlapping with LINE1 annotation). Some of the SVs in this category had clear signals of copy number changes around the breakpoints (Supplementary Figure S8), giving another evidence that they were genuine somatic SVs.

Breakpoint positions detected by nanomonsv on ONT sequencing data were mostly (96.7%) within two bp of those detected by Illumina sequencing data (Figure 3C), despite the difference in error rate between the two platforms. Therefore, reasonably accurate identification of breakpoint positions is possible with error correction and careful examination of supporting reads from error-prone long-read sequencing.

Ninety-nine somatic SVs were those affecting known cancer-related genes (46). These included important cancer genes such as the 12 kb deletion of *PTEN* in COLO829 (47) and the 5kb deletion of *STK11* in H2009 though these were also identified by the short-read platform.

### Evaluation of nanomonsv using benchmark dataset and simulation

We compared 49 somatic SVs obtained by nanomonsv using ONT sequencing data of COLO829 with high-confidence somatic SV sets for the same cell-line generated by high-coverage short-read platforms and multiple variant callers (35) (Arora benchmark hereafter) as well as multi-platform combined with extensive experimental validation (36) (Valle-Inclan benchmark). Among 75 and 58 somatic SVs by Arora and Valle-Inclan benchmark, nanomonsv detected 44 and 46 SVs (Figure 3D, Supplementary Figure S9a). Assuming that novel SVs by nanomonsv (6 and 4 SVs, respectively) were all false positives, the ratios of precision were 87.8% (43/49) and 91.8% (45/49), and recall was 57.3% (43/75) and 77.6% (46/58) at worst (Figure 3E). This tendency was robust when we applied nanomonsv to higher-depth Nanopore sequence data (sequence yield, tumor: 190.12 Gbp, normal: 138.80 Gbp) and PacBio sequencing data (sequence yield, tumor: 137.16 Gbp, normal: 145.28 Gbp) from the same cell-line and their matched control deposited as PRJEB27698 (36) (Figure 3D, E, and Supplementary Data 4). Although the recall was slightly lower for Arora benchmark, the number of supporting reads for their sequence data was generally small (Supplementary Figure S9b).

To evaluate the importance of the approach jointly utilizing tumor and matched control samples, we separately applied regular SV detection tools (Sniffles2 (37), cuteSV (15), Delly (38) and SVIM (13)) to tumor and matched control samples with different thresholds, and filtered out the SVs called in matched controls from those in tumors (we call this approach as ‘separate detection and subtraction approach’). The precision and recall of this approach were inferior to those of nanomonsv, suggesting that simultaneously utilizing tumor and matched control data is effective for the sensitive and accurate identification of somatic SVs (Figure 3F, Supplementary Figure S9c). We have also evaluated the software CAMPHORsomatic (39), which handles tumor and matched control samples simultaneously. The precision and recall of nanomonsv were better than those of CAMPHORsomatic (Figure 3F, Supplementary Figure S9c). Next, we evaluated the performance of nanomonsv using simulation data with different tumor purity and sequence yields. Overall, the precision and recall of nanomonsv were superior to other approaches. Although the recall ratio became small for very low tumor purities and sequence yields, precision was relatively stable, implying the robustness of nanomonsv (Supplementary Figures S10–S12). Especially in the case of low tumor purity, the sensitivity is significantly reduced without a decent sequence yield. Therefore, even for long reads, it is desirable to have 30–40× coverage (roughly equivalent to 90–120 Gbps yield) as in typical short-read-based studies (48).

### Characterization of mobile element insertions

**Figure 4.**
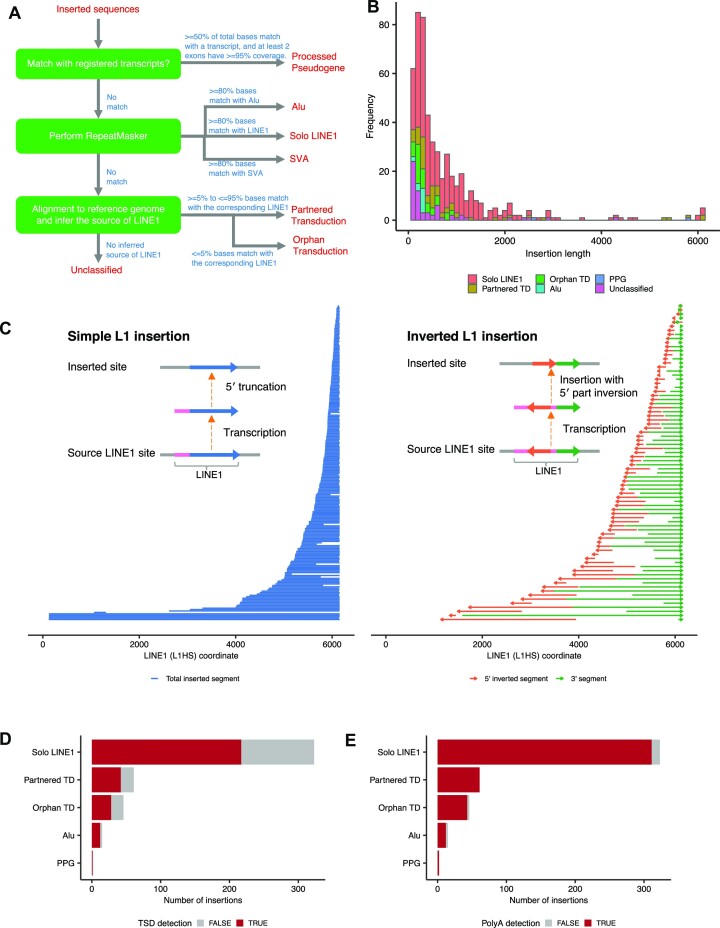
Classification and structure of inserted sequences between somatic SV breakpoints. (**A**) A simplified chart for classifying inserted sequences used in this study. See the Materials and Methods for detail. (**B**) The size and classification distribution (histogram in bins of 100 bp) of inserted sequences. Partnered TD, Orphan TD and PPG are partnered transduction, orphan transduction and processed pseudogene, respectively. (**C**) Diagram showing the position of each solo LINE1 inserted sequence without (left) and with (right) 5′ inversion within the human-specific LINE1 sequence (L1HS). The horizontal lines or arrows in the same vertical position show single solo LINE1 insertion events. They mostly start from the middle (by 5′ truncation) but usually end at 3′ end of LINE1. (**D**, **E**) The number of insertions with detected target site duplications (TSDs) and polyA tails stratified by the categories of inserted sequences.

Canonical SV module identified a total of 509 insertions, among which 492 were from H2009. For insertions, our approach can identify complete inserted sequences as well as inserted positions. There are many possible types of insertions, such as tandem duplication, mobile element insertions (MEIs), viral sequence integration, and processed pseudogene. To systematically characterize the inserted sequences, especially focusing on MEIs, we have developed a pipeline for classifying the inserted sequences based on comparison with transcriptome, annotation with repeat sequence information, and re-alignment to the reference genome (see Figure 4A).

First, if the inserted sequence significantly matched with a transcript, the insertions were classified into processed pseudogene (49,50), which are copies of mRNAs integrated into the genome by reverse transcriptase activity of LINE1 elements. We identified two processed pseudogenes affecting *IBTK* and *CARNMT1* genes in H2009 (see Supplementary Figure S13). Although the existence of these pseudogene insertions had been identified by the short-read platform using the same cell-line (49), a detailed structure of the entire inserted sequence such as the position of the inversion breakpoint was first confirmed in this study.

Second, when either of three major mobile elements (LINE1, Alu and SVA) covered most of the inserted sequence (≥80% by RepeatMasker, http://www.repeatmasker.org), the inserted sequences were categorized into each class. We identified 323 LINE1 and 15 Alu insertions in three cell-lines, respectively (Figure 4B). The LINE1 insertions are frequently accompanied by inversion at the 5′ end, whose mechanism can be explained by ‘twin priming’ (51). In fact, by investigating inserted sequences, the 5′ inversions were observed in 81 (25.1%) of LINE1 insertions. In addition, 5′ inversions were frequently accompanied by the partial loss of internal LINE1 sequences, which might occur during the integration process (Figure 4C). We also observed other complex structural changes. One example was 1100 bp insertion at chromosome 14, which was a direct concatenation of 160 bp 5′ end and 900 bp 3′ end LINE1 sequence without a 5′ inversion. These diversities of insertion structures produce some deviations between inferred insert sequence lengths from short-read and long-read sequence data (Supplementary Figure S14) because accurate inference of the insert nucleotide length from short-read sequencing data is difficult.

Next, the remaining insertions were aligned to the human genome to explore LINE1 3′ transductions, in which unique DNA segments downstream of LINE1 elements are mobilized as part of aberrant retrotransposition events (52). Transposed sequences can be a combination of LINE1 elements and their downstream sequences (partnered transductions) or only downstream ones (orphan transductions). When a LINE1 element existed upstream of the aligned site of inserted sequences, we can infer that the LINE1 element is the source of transduction. As possible LINE1 sources, we first extracted 5228 full-length evolutionarily recent primate-specific LINE1 elements from the human reference genome (reference putative LINE1 source elements). In addition, since several active non-reference LINE1 source elements can be detected as polymorphic insertions, we also included 652 and 2610 full-length LINE1 insertions identified in 1000 genomes Phase 3 (53) and gnomAD v2.1 (54), respectively. Furthermore, when many inserted sequences were aligned to the same genomic locations, we searched for the germline LINE1 insertion near those positions from the normal sequence data and manually curated the putative rare germline LINE1 insertions that were considered as the sources of LINE1 3′ transduction.

**Figure 5.**
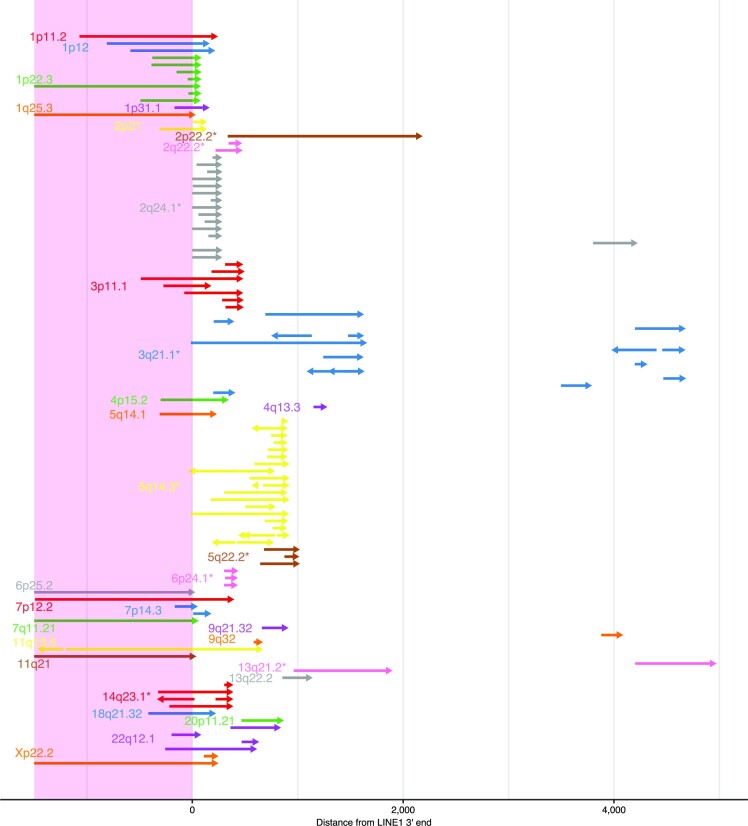
A comprehensive picture of L1 transductions identified in H2009. Horizontal arrows in each vertical position show distinct LINE1 transduction events whose corresponding LINE1 source sites are distinguished by color and labeled by cytoband. Asterisks beside the labels indicate that the source sites are not in the human reference genome. Arrows starting before the position of LINE1 3′ ends (within LINE1 sequences shaded by light pink) are partnered transductions, whereas those starting after LINE1 3′ ends are orphan transductions. Multiple arrows in one line indicate some structural changes in the inserted sequences (most typically internal inversions depicted by two outwardly directed arrows) .

**Figure 6.**
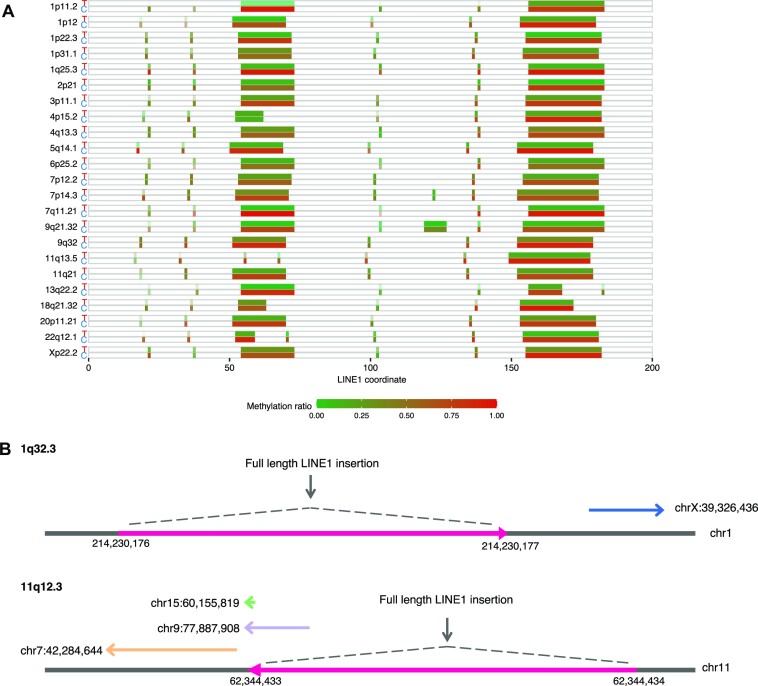
Characterization of L1 transductions identified in H2009. (**A**) Methylation status of promoters of somatic LINE1 source elements for H2009. For each LINE1 source site (labeled by cytoband), the upper and lower boxes represent the tumor (T) and matched control (C) methylation states. After the detection of methylated bases for each CpG site using nanopolish, the ratios of methylations were calculated. Contrasting density was determined by the depth of sequence covering each site. *P*-values measuring the significance of methylation frequency difference between the tumor and match control at each source element ranged from $1.17 \times {10^{ - 62}}$ to $1.37 \times {10^{ - 6}}$ with a median of $4.43 \times {10^{ - 13}}$ (see Materials and Method for detail). (**B**) Examples of nested LINE1 insertion identified in H2009. Two full-length LINE1 insertion sites became the new active sources of LINE1 transductions. The novel source site at 1q32.3 generated one orphan LINE1 transduction. The second novel source site at 11q12.3 eventually produced two partnered transductions and one orphan transduction.

We identified 107 somatic 3′ transduction events (61 partnered and 46 orphan transductions) from 33 putative LINE1 source elements, of which 105 from 31 source elements were from H2009 (Figure 5). Of the 24 LINE1 sources from the reference genome, 20 belonged to the human-specific LINE1 (L1HS) subfamily, three to L1PA2, and one to L1PA4 (the second and fourth youngest primate-specific subfamilies, respectively). Nine were derived from non-reference LINE1 source elements (four from 1000 Genome Phase 3, three from gnomAD, and two from manual curation), corroborating the importance of population- and individual-specific hot LINE1 elements (55). Several transductions included the 5′ inversions, implying the same mechanism as solo L1 insertion, such as twin priming functions during reverse transcription. For each LINE1 source element, 3′ end positions of the inserted sequences tended to concentrate at the close genomic positions. This may be because these 3′ end positions are probably the location where the transcription is terminated, and the positions with a potency of transcription termination may be scattered because they require some characteristic sequences. As localized hypo-methylation of the LINE1 promoter region has been reported to drive the somatic activation as source elements (20), we quantified the methylation level using nanopolish (56) on raw signal-level data of ONT sequencing. For all the 23 reference LINE1 source elements, the methylation ratios were significantly lower in tumors than in the matched controls (Figure 6A, Supplementary Figure S15). We also identified two examples of nested LINE1 transduction (20), where somatically inserted LINE1 elements themselves became the source of the next LINE1 transduction (Figure 6B).

The refinement step of the nanomonsv procedure performs error correction of the insert sequences. The accuracy of the insert sequences by nanomonsv was estimated to be mostly more than 95% (Supplementary Figure S16a). This refinement of inserted sequences enabled us to investigate the features such as target site duplications and polyA tails, which were frequently accompanied by MEIs (Supplementary Figure S16b). Target site duplications and poly-A tails were observed in 67.2% (314/467), and 96.8% (452/467), respectively (Figure 4D, E). These results suggest that long-read sequencing has great potential for characterizing various mechanisms of genomic insertions.

### SVs connected with centromere and telomere sequences

Single breakend SV module identified in a total of 91 somatic single breakend SVs (3, 38 and 50 in COLO829, H2009 and HCC1954, respectively, see Supplementary Data 5, 6). Of those, 32 single breakend SVs were bound to satellite (23 and 5 SVs for alpha satellite and human satellite sequences, respectively) or simple repeat sequences (4 SVs). Although even short-read sequences can be used to identify single breakend SVs with satellite or simple repeat sequences (28), long-read sequencing enables us to elicit more refined information about their nature by assembling the raw read after the breakpoint.

**Figure 7.**
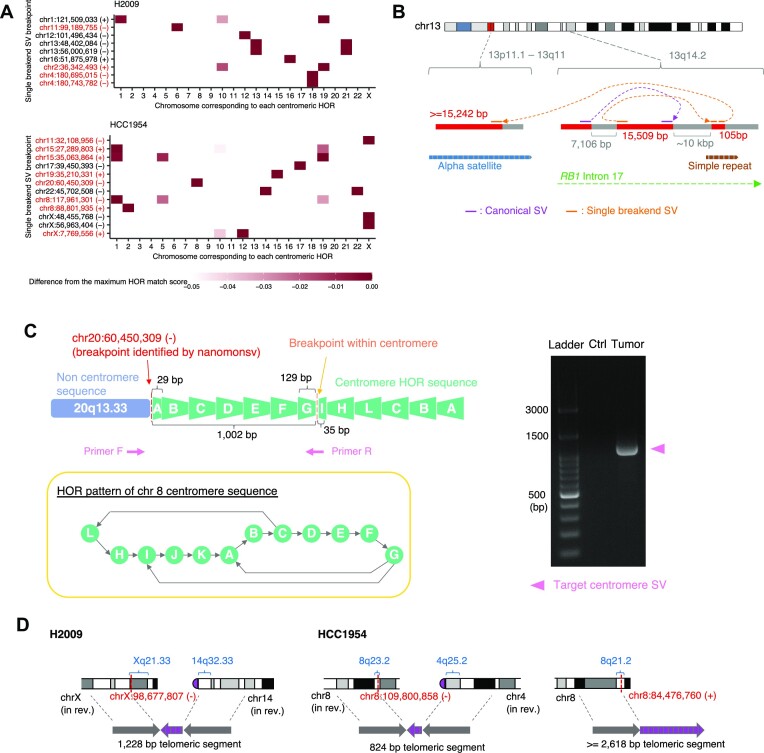
Single breakend SVs involving alpha satellite and telomere sequences. (**A**) For each single breakend SV (whose breakpoint was illustrated as chromosome:position (direction) in the axis label) linked to alpha satellite sequence, heatmaps depict the consistency of the contig sequence with the respective centromere HOR for each chromosome. The color intensity of cells was determined by the deviation from the maximum HOR score across HORs within each SV. Single breakpoint SVs that were considered to be interchromosomal were shown in red. (**B**) Example of complex SVs involving centromere sequence affecting *RB1* gene in H2009. The inversion within *RB1* gene (colored by purple) was identified by Canonical SV module. Single breakend SV leading to the alpha satellite region via 105 bp segment, whose exact location of the 105 bp segment could not be identified because it matched to several positions in a simple repeat region, was identified by Single breakend SV module. See also Supplementary Figure S19. (**C**) A characteristic example of single breakend SV connected to an alpha satellite sequence accompanied by inversion in the vicinity of the breakend on the alpha satellite side. This SV could be validated by PCR because we were able to design a pair of primer sequences both of which straddle the cancer-specific breakpoints, and the product size was modest (∼1000 bp). See also Supplementary Figure S18. (**D**) SVs involving telomere sequences identified by Single breakend SV module. Some karyotypes were placed in reverse (in rev.).

In alpha satellite regions, various types of approximately 171 bp monomer sequences constitute high order repeat (HOR) structure per centromere region (44). In chromosome X, 12 divergent monomers are ordered to form an approximately 2000 bp canonical HOR (ABCDEFGHIJKL), which occupies most of the centromeric region over millions of bases (57). On the other hand, non-canonical forms of HOR structures specific to populations and individuals are occasionally observed (58,59). For each of the 21 single breakend SVs leading to alpha-satellites (excluding two that matched inactive alpha-satellite sequences), we examined the consistency of the contig sequence with the HOR pattern at the centromere of each chromosome by calculating the HOR match score (Figure 7A, see Materials and Methods for details). At least, 12 single breakend SVs were estimated to be interchromosomal (Supplementary Data 6), and seven of them corresponded to the derivative chromosomes inferred by previous SKY karyotype experiments (resource hosted on the Cellosaurus website (60,61)). Also, we could validate 7 out of 8 using PCR (see Supplementary Figure S17). Therefore, translocation involving centromere sequences may be a frequent event.

Most of the estimated HOR from the contig centromere sequence were canonical ones which are chromosome-specific and evolutionary defined (44). On the other hand, we identified non-canonical HORs in three single breakend SVs bound to alpha satellite sequences. One single breakend SV at chromosome 11 connected to the centromere sequence of chromosome X had a 17-mer monomer of ABCDEFGHIJKLHIJKL (Supplementary Figure S18).

We detected a single breakend SV joining a centromere sequence of chromosome 13 and complex rearranged regions in *RB1*, a well-characterized tumor suppressor gene located in the region distant from the centromere sequences (Figure 7B, Supplementary Figure S19). Furthermore, we identified a single breakend SV at chromosome 20 connected to chromosome 8 alpha satellite sequences with an inversion in the alpha satellite side near the breakpoint, which was validated by PCR (Figure 7C). We have also identified three single breakend SVs leading to telomeric sequences (Figure 7D, Supplementary Figure S20) (62,63), two of which, corresponding derivative chromosomes have been detected by previous SKY (der(14)t(X;14) in H2009 and der(2)t(2;8;4) in HCC1954) (60,61). These observations suggest that SVs involving centromere and telomere sequences are common events in cancer, and our approach can help reveal their complex structures.

### LINE1-mediated rearrangements detected by single breakend SV module

Many contig sequences of single breakend SVs showed prominent patterns indicative of LINE1-mediated rearrangement, where the first portion matched the LINE1 sequence and the remaining portion unambiguously matched the human genome sequence distant from the breakpoints (Supplementary Figure S21). Although its presence is widely known, LINE1-mediated rearrangement has been notoriously difficult to detect from short-read sequencing data.

**Figure 8.**
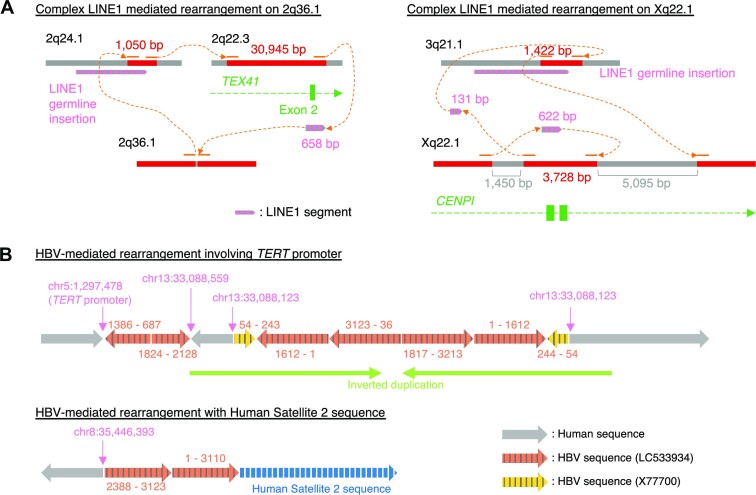
Complex LINE1- and HBV-mediated rearrangements identified by nanomonsv Single breakend SV module. (**A**) Examples of complex SVs with multiple LINE1-mediated rearrangements as components. (**B**) Examples of complex HBV integrations. The number pairs listed on the side of each HBV segment indicate the start and end coordinates in the HBV sequences (LC533934 and X77700).

In the H2009 cell line, where LINE1-mediated deletions were analyzed extensively in previous studies using a short-read platform (20,21). Our analysis detected 12 LINE1-mediated deletion and rearrangement events. Ten of these were accompanied by local deletions (112–10430 bp), of which six had also been detected in previous studies. The newly detected ones tended to have shorter inserted LINE1 sequences. We also newly identified one large intrachromosomal rearrangement and one interchromosomal translocation mediated by LINE1 sequences. Three newly identified LINE1-mediated rearrangements were validated by PCR (Supplementary Data 7). Most LINE1-mediated SVs had a relatively simple structure where different locations were connected via LINE1 segments. However, we also identified two complex LINE1-mediated rearrangements (Figure 8A, Supplementary Figure S22). One was predicted to be an insertion with approximately 30 000 bp in length from a distant genomic region, mediated by a 658 bp LINE1 segment and an orphan transduction. The other was an inversion event affecting the *CENPI* gene with two breakends, one of which was derived from a partnered transduction from a non-reference LINE1 source site on 3q21.1. In the HCC1954 cell line, we also identified one interchromosomal translocation mediated by a LINE1 and one putative Alu-mediated deletion (Supplementary Figure S23). While poly-A tails were observed in the majority of LINE1-mediated rearrangements (10 out of 12), no rearrangements had target site duplications, consistent with previous studies (21,64).

### Hepatitis B virus integration detection

Viral integration into the cancer genome is fairly frequent in cancers such as human papillomavirus (∼8000 bp) in multiple cancers (65), hepatitis B virus (HBV) (∼3300 bp) in liver cancers (66) and human T-cell leukemia virus type I (∼9000 bp) in adult T-cell leukemia/lymphoma (67). We have applied nanomonsv to a cell-line, PRC/PRF/5, known to have HBV integration. Since there were no matched controls for this cell-line, we used BL2009 cell-line as a dummy matched control and just focused on HBV integration detection. We identified 12 HBV integrations. Most of these integrations were identified by Single breakend SV module because the integrations were usually accompanied by large deletions and translocations. Nanomonsv identified not only all the integrations identified in previous studies by Illumina short-read platforms but also one new integration (Supplementary Data 8). However, the advantage of long-reads is the ability to reconstruct the HBV insertion site and internal sequence completely. We observed that one integration had characteristic inverted duplication consisting of HBV and human genome sequences around the integration sites. For example, in the HBV-mediated rearrangement that connected the *TERT* gene promoter (known as the frequent HBV integration site (66,68,69)) on chromosome 5 and to the locus of chromosome 13, intermittent segments of human and viral sequences formed inverted duplication (Figure 8B). Furthermore, we identified an HBV-mediated rearrangement at chromosome 8 connected to a Human Satellite 2 sequence, whose origin was predicted to be the one in chromosome 1 by alignment to the CHM13 reference genome (70), suggesting that this event is an HBV-mediated interchromosomal translocation.

## DISCUSSION

We proposed two approaches for identifying somatic structural variations (SVs), Canonical SV module and Single breakend SV module. Canonical SV module can identify the majority of the SVs identified from short-read platforms as well as novel ones. The precision and recall of Canonical SV module were demonstrated to be superior to the ‘separate detection and subtraction approach’ using existing SV detection tools. Furthermore, we have developed a workflow for detecting and classifying single breakend SVs (Single breakend SV module). We demonstrated that it could identify complex SVs, such as those involving satellite sequences, LINE1-mediated rearrangement, and viral integration, which had been difficult to detect by short reads.

We could determine the breakpoints of SVs with a single-nucleotide resolution with non-templated sequence insertions to some extent. Currently, most sophisticated algorithms on short-read platforms support single-nucleotide resolution detection using split-read evidence or local assembly. However, there has been little evaluation on the resolution of breakpoints of SVs using noisy long-read sequencing data. Identifying breakpoints at single-nucleotide resolution allows us to identify micro-homology and non-templated sequence insertions, which can provide us with valuable information about the mechanisms that generate SVs (71,72). In addition, it is highly preferable for comparison and annotation with SVs registered in a public database.

In this paper, we did not focus on somatic VNTR/microsatellite repeat expansion events (73). Although long-read sequencing technology can potentially improve the detection of repeat expansion events, the current framework based on the reference genome may not be appropriate to capture long repeat expansion events because the reference genome is not reliable at the location susceptible to these events. One possible approach to capture these events may be to list microsatellite and VNTR regions beforehand, count the number of repeats using short tandem repeat aware alignment algorithms, and measure the difference in repeat count profiles between tumor and matched control data.

Although the current approach successfully identified somatic SVs and MEIs, detection of those present in the minority of cells (subclones) is still challenging with a modest sequencing depth. One way to deal with this is to perform target region amplification by adaptive sampling (74,75). Another possibility to tackle this problem would be to combine single-cell sequencing technologies (76) with long-read platforms.

On the other hand, the interpretation of the detailed structure and properties of complex SVs is not fully automated at present, and much of the work is done manually, which remains a challenge for processing many samples. For this purpose, there is a need to cover and classify more ‘complex’ forms of SVs. In addition, visualization methods need to be developed to facilitate interpretation. It will also be necessary to establish an appropriate format for describing complex SVs in the future.

Single breakend SV module incorporates some assembly. However, it cannot detect SVs where both of the breakpoints are located in areas where reference genomes are not well-characterized, such as highly repetitive regions. It will be necessary to obtain and utilize a complete reference genome for each individual (70) or consider using a graph genome that covers a major variation of human genomes (30).

## Supplementary Material

gkad526_Supplemental_Files

## Data Availability

The raw Oxford Nanopore sequence data and Illumina short-read sequence data used in this study are available through the public sequence repository service (BioProject ID: PRJDB10898).
