# Supplementary material for: Precise characterization of somatic complex structural variations from tumor/control paired long-read sequencing data with nanomonsv
Source: Nucleic Acids Res. 2023 Jun 20;51(14):e74. doi: 10.1093/nar/gkad526 (PMC10415145; doi:10.1093/nar/gkad526)
Supplement: gkad526_Supplemental_Files [file gkad526_supplemental_files.zip › SupplementaryInfomation.pdf]

Supplementary Information

Precise characterization of somatic complex structural variations from tumor/control paired long-read sequencing data with nanomonsv

Yuichi Shiraishi, Junji Koya, Kenichi Chiba, Ai Okada, Yasuhito Arai, Yuki Saito, Tatsuhiro Shibata, Keisuke Kataoka

Supplementary Text

Detailed algorithm of nanomonsv

In nanomonsv, canonical SVs (where two breakpoints are identified) are divided into three categories according to how these SVs are supported by each read. We denote the SVs that are supported by single alignment with insertion or deletion (‘I’ or ‘D’ of CIGAR strings) as “I-type” and “D-type,” respectively. SVs that are represented by multiple alignments (primary alignment and one or more supplementary alignments) that are consecutive when viewed from the query sequence are denoted as “R-type.” In addition, single breakend SVs, which are supported by alignments with soft clipping (‘S’ of CIGAR strings) are denoted as “S-type.” Please note that the procedure of each step becomes slightly different depending on these types. The soft-clipped part of S-type SV may or may not be aligned to other genomic positions. Therefore, some of S-type SVs are expected to overlap with the R-type SVs. They are identified in independent procedures, but integrated at the final step.

Parsing step

Parsing I-type and D-type SV supporting reads

For putative I-type and D-type SV supporting reads, we parse a CIGAR string of each alignment of the input BAM file to collect the information such as chromosome, indel start, and end positions, putative indel size, and read IDs and organized as BED file. Then, the records are sorted by the genomic coordinate and bgzip’ed and tabix’ed (http://www.htslib.org/).

Parsing R-type SV supporting reads

In order to gather R-type supporting reads, we search for multiple “consecutive alignment” of a single read, in which the query end of one alignment is in close proximity (within 50 bp) to the query start of the next alignment, and thus the corresponding genomic coordinates become the breakpoint of putative SVs. First, by parsing the input BAM file, query start and end positions and target (genomic) start and end positions, as well as alignment directions, are collected for each read ID and alignment (primary and supplementary alignments not including secondary alignments). Then, for each read ID, we find the “consecutive alignment,” and the possible ranges (± 30 bp margin from the corresponding genomic coordinates) of the two genomic breakpoints of putative SVs, breakpoint direction and read ID are recorded and organized as BEDPE format. Then, these records are sorted by genomic coordinates and bgzip’ed and tabix’ed.

Parsing S-type supporting reads

We examine genomic coordinates and direction of breakpoint as well as the soft-clipped sequences, and arrange them to be in BED format. Then, the records are sorted and bgzip’ed and tabix’ed. In addition to supporting reads for S-type SVs, these S-type supporting reads are also used to supplement the evidence for

I- and D-type SVs in cases where I- and D-type supporting reads cannot cover the entire I- and D-type variant (such as insertions) and are aligned with soft-clipping.

Clustering step

For each of two R-type SV supporting reads, when both the possible ranges of the breakpoints overlap, they are merged so as to support the same SV. For I-type and D-type supporting reads, when the possible ranges of the indels overlap and the size of the indel is about the same (within 20%), the two supporting reads are merged. For S-type supporting reads, when the possible ranges of the breakpoints overlap and the directions of the breakpoints are common, they are merged. For each type of supporting read, the merging procedure is repeated until there is no pair of supporting reads to be merged.

For each cluster, after adding the breakpoint supporting reads nearby, we remove those having in total less than three supporting reads or a median of <= 40 mapping qualities. Also, if there are apparent supporting reads (the breakpoints are within 50 bp from any of the supporting reads in the cluster, and for indels, the size is between 75 ~ 125% of the median of the indel size in the supporting reads) for the putative SVs in the matched control sample, these SVs are removed.

Here, we also filtered out putative SVs using the control panel (which is supported from nanomonsv version 0.5.0). We performed the parse step for 30 Nanopore sequencing data from the Human Pangenome Reference Consortium (Wang et al. 2022) beforehand, and they are merged into one BEDPE or BED file via “nanomonsv merge” command. If supporting reads for the putative SVs in the control panel samples, these putative SVs were removed from the candidate.

Refinement step

Consensus sequence generation

First, for candidate SVs, we extract the part of supporting reads around the breakpoint. For D-type and R-type SVs, 300 bp sequences before and after the position corresponding to SV breakpoints within the supporting reads are extracted. For I-type SVs, the entire inserted sequences as well as 300 bp from both ends are derived for the supporting reads. Next, for each SV, we perform error correction to generate the consensus sequence. First, we select a representative supporting read for a template sequence (here, breakpoint supporting reads are excluded from the selection because they may not cover the entire variants). Next, we perform pairwise alignment using the parasail library (Daily 2016) for each supporting read with the template sequence to generate PAF format file. Then, we perform racon (Vaser et al. 2017) to obtain the first-round error-corrected sequence. Then, setting this first-round error-corrected sequence as the next template sequence, we perform the same procedure to generate the second-round error-corrected sequence, which is adopted as the final consensus sequence.

For candidate S-type SVs, we extract 300 bp sequences before the breakpoint and entire sequences after the breakpoints are extracted. Then, after performing all vs all alignment using minimap2 with “-x ava-ont” option, we select the one that contains the most other reads and set it as the template read. The error-corrected consensus reads are then obtained in the same manner as above, but this time minimap2 with “-x map-ont” option is used as the tool for pairwise alignment.

SV breakpoint coordinate determination

A one-time jump Smith-Waterman (OJ-SW) algorithm, where one query sequence is compared with the two target sequences (starting from the target sequence 1 and switched to the target sequence 2 at some point, see Supplementary Figure 1) is used to determine the coordinates of breakpoints and inserted sequences within them for I-, D-, and R-type SVs. For each D-type and R-type SV, the two sequences around the regions where the possible locations of the first and the second breakpoints are extracted from the human reference genome sequence and are used as the target sequences 1 and 2 for the OJ-SW algorithm, respectively. The consensus sequence generated in the above step is set as the query sequence. After performing the OJ-SW algorithm, the two genomic coordinates corresponding to where the jump from the target sequence 1 to 2 occurred are determined to be the SV breakpoints, and the skipped query sequence by the leap is set as the inserted sequence between the breakpoints. For each I-type SV, the sequences around the putative insertion start and end positions within the produced consensus sequence are set as the target sequence 1 and 2 for the OJ-SW algorithm, respectively. For the query sequence, the sequences around the region where the insertion is considered to be located are extracted from the reference genome. Then, after performing the OJ-SW algorithm, the position where the jump occurred within the query sequence is set to be the exact coordinate of insertion, and the skipped sequences are set to be the deleted nucleotides. In addition, the points where the jump happened in the target sequences are set as the start and end of inserted bases.

For S-type SVs, an ordinary Smith-Waterman algorithm is used to determine the coordinate of breakpoints. A sequence around the possible breakpoint locations is extracted from the human reference genome sequence and

Validation step

For each SV candidate, constitute the putative SV segment sequence by concatenating 200 bp sequences from both the breakpoints. When there is an inserted sequence, two SV segment sequences are prepared: For each breakpoint, we prepare 200 bp sequences from one breakpoint joined to the 200 bp sequences in the opposite direction including the inserted sequence and subsequence nucleotides after the other breakpoint (if the size of insertion is below 200bp). Therefore, putative SV segment sequences are 400 bp in size. Then, after collecting the Nanopore reads spanning the SV breakpoint from both tumor and matched control samples, local alignments of SV segments sequences to each read are performed using the parasail library (Daily 2016). We set match, mismatch, gap opening, and gap extension scores as 2, -2, -3, and -1, respectively. Here, in order for each Nanopore read to be an “SV variant read” (the read containing the SV segment sequence), we request that the alignment score (we adopt the larger score between the two SV segment sequences in case there is an inserted sequence) is equal or more than 560. Then, we count the number of SV variant reads for tumor and matched control samples and keep the SVs whose SV variant reads equal or more than 3 for the tumor and zero for the control samples.

Classification and characterization of the inserted sequences

Check for processed pseudogene

First, the inserted sequences of insertion SVs are aligned to the human reference genome using minimap2 with the Nanopore 2D cDNA-seq option, "-ax splice." Then, for each alignment, the intersection with the exonic regions by comprehensive gene annotation set from GENCODE version 31 is investigated. If there exists a transcript in which more than one exon matches >= 95% and >= 50% of the inserted sequence matches a transcript, then this insertion is determined to be a processed pseudogene.

Detection of target site duplications and polyA tails

If there is a sequence of 10 or more consecutive characters of A in 30 bp from the beginning of the inserted sequence, or a sequence of 10 or more consecutive characters of T in 30 bp from the end of the sequence, we recognize that the inserted sequence has a polyA tail. If either of the following conditions is met, then we recognize that target site duplication exists.

(1) Twenty bp from the beginning of the inserted sequence is aligned to the 20 bp from the left of the genomic insertion site with Smith-Waterman algorithm. Then, alignment starts within two bp from the start of the inserted site, has five or more matched sites, and has an identity ratio is 80% or more.

(2) Twenty bp from the end of the inserted sequence is aligned to the 20 bp from the left of the genomic insertion site with Smith-Waterman algorithm. Then, alignment ends within two bp from the end of the inserted site, has five or more matched sites, and has an identity ratio is 80% or more.

Check for Alu, Solo LINE1, SVA insertion

For the remaining insertions, we perform the RepeatMasker with "-species human" option. Then, the portions of bases annotated as LINE1 ("LINE/L1"), Alu ("SINE/Alu"), or SVA ("Retroposon/SVA") among the total nucleotides subtracted by the parts annotated as poly-A or poly-T ("(T)n" or "(A)n") are calculated. When either of them is equal or more than 80%, then the insertion is classified into those categories.

Check for partnered or orphan transduction

First, we created a database of possible sources of LINE1 transduction. For LINE1 included and annotated in the human reference genome, we downloaded RepeatMasker file (http://hgdownload.soe.ucsc.edu/goldenPath/hg38/database/rmsk.txt.gz) and selected the records whose family is L1, whose subfamily is among those of recent primate-specific ones (L1HS, L1PA2, L1PA3, L1PA4, and L1PA5), and whose size is equal or larger than 5,800, resulting in 5,228 records. Then, for those not included in the reference genome (and thus the polymorphism of LINE1 insertion), we obtained 1000 genomes Phase 3 SV file (ftp://ftp.1000genomes.ebi.ac.uk/vol1/ftp/phase3/integrated_sv_map/ALL.wgs.mergedSV.v8.20130502.svs.genotypes.vcf.gz) and filtered them by "bcftools filter" command (https://github.com/samtools/bcftools) with "INFO/SVLEN > 5800 && INFO/SVTYPE == 'LINE'" option, remaining 652 records. Also, we extracted

gnomAD v2.1 SV file (https://storage.googleapis.com/gnomad-public/papers/2019-sv/gnomad_v2.1_sv.controls_only.sites.vcf.gz) and selected near full-length LINE1 polymorphisms by “bcftools filter” command with the “ALT == ‘<INS:ME:LINE1>’ && INFO/SVLEN >= 5800” option. Since these 1000 genomes and gnomaAD SV files are based on the hg37 reference genome, we converted the coordinates using liftOver (Kuhn, Haussler, and Kent 2013) to the hg38 coordinate system. Then, all the records were merged into one bed file and bgzip’ed and tabix’ed.

For each inserted sequence, alignment is performed using BWA-MEM (Li 2013). Then, we checked whether the primary alignment has >= 30 mapping quality and any records of possible LINE1 source databases constructed above within 5,000 bp. If these requirements are not met, then the inserted sequence is classified into “Other.” When these are satisfied, we set the proximal record as the corresponding LINE1 source element for the transduction, and we extract all the supplementary alignment that is within 5,000 bp of the primary alignment for possible inversion. Then, by the portion of bases annotated as LINE1 by RepeatMasker, the insertion is classified into Orphan transduction if the ratio is below 0.01, or Partnered transduction otherwise.

Investigation of error ratios of inserted sequences inferred by nanomonsv

The accuracies of inserted sequences inferred by nanomonsv are measured by aligning inserted sequences of LINE1 transductions to the reference genome and investigating the matched parts. The accuracy is defined as the number of matched bases divided by the summation of the numbers of matched and mismatched bases plus the total sizes of all the insertions and deletions in the alignment (Rang, Kloosterman, and de Ridder 2018).

Classification of single breakend SVs

Preprocessing for single breakend SV classification

First, we make a list of all breakpoints (two for one SV) for all SVs detected by Canonical SV module. Then, for each single breakend SV, if there is a breakpoint in the above list that matches the chromosome, direction and genomic coordinate (margin of up to 50bp allowed), it is removed. Then, for each contig of the remaining single breakend SVs, we perform alignment to the human reference genome by BWA-MEM (Li 2013) version 0.1.17 with the option “-h 200” and. Also, we perform RepeatMasker with “-species human” option.

Rescuing canonical SVs

When the contig of a single breakend SV has a >= 2000 bp segment in the vicinity of breakpoint (< 100 bp) that is aligned to the human genome reference with >= 40 mapping quality, then Single breakend SV is reclassified into a canonical SV.

High repeat single breakend SVs

Next, if the majority (>= 80%) of the single breakend contig is annotated as either “Simple_repeat”, “Satellite”, or “Satellite/centr” by RepeatMasker, they are categorized into the High repeat single breakend SV.

LINE1-mediated rearrangement

Segments by alignment to the human reference genome sequence by BWA are sorted in ascending order by the coordinates of the query (single breakend contig) start. Then, for the first segment of the above genome alignment or the first and second segments combined, if either of the following two conditions is satisfied, Single breakend SV corresponding to the single breakend contig is determined to be LINE1-mediated rearrangement.

1. The size of the next genome alignment segment is equal or greater than 2000 bp and the mapping quality is equal or greater than 40.
2. The size of the next genome alignment segment is greater than 2000 and the corresponding target position (human genome reference sequence) is the same chromosome as the breakpoint of Single breakend SV and within 10,000 bp.

Finally, single breakend SVs that are not canonical SVs, high repeat single breakend SVs, or LINE1-mediated rearrangements are labeled as "unclassified."

Structural variation detection from short-read sequencing data

GenomonSV

GenomonSV (https://github.com/Genomon-Project/GenomonSV) version 0.7.2 was used. First, “GenomonSV parse” command was performed for both tumor and matched control BAM files. Then, “GenomonSV filt” was performed on the tumor data with the options “--min_junc_num 2”, “--min_overhang_size 30”, and “--max_control_variant_read_pair 10” with specifying the matched control BAM file for the “--matched_control_bam” option. Then we performed additional filtering with sv_utils filter, custom software for post-processing GenomonSV results (https://github.com/friend1ws/sv_utils), with “--min_tumor_allele_freq 0.07”, “--max_control_variant_read_pair 1”, “--control_depth_thres 10”, and “--inversion_size_thres 1000” options.

Manta

We used manta (https://github.com/Illumina/manta) version 1.6.0. First, we performed configManta.py with the default options and runWorkflow.py for each tumor and matched control pair with “-m local,” and “-j 8” options. Then, we extracted records tagged with “PASS” in the FILTER columns using “bcftools view” command.

SvABA

SvABA (https://github.com/walaj/svaba) version 1.1.0 was used. First, we performed “svaba run” command for each tumor and matched control data using “-p 8”, “-v 1 -A” options. Then, we performed filtering by “bcftools

view” command with the “-f PASS” option and “bcftools filter” command with the “'FORMAT/AD[0:0]<=1&&FORMAT/AD[1:0]>=2&&FORMAT/DP[0:0]>=10&&FORMAT/DP[1:0]>=10” option.

GRIDSS

We used GRIDSS (https://github.com/PapenfussLab/gridss) version 2.8.0. First, “gridss.sh” was performed on tumor and control pairs with “-j gridss-2.8.0-gridss-jar-with-dependencies.jar”, “-t 8”, and “--picardoptions VALIDATION_STRINGENCY=LENIENT” options. Then “gridss_somatic_filter.R” was performed with the default option. Then we used the “bcftools view” command with “-i INFO/MATEID[0]!=’’ and “-f PASS” options.

TraFic-mem

First, since TraFic-mem currently only supports GRCh37-based BAM files, we aligned the short-reads to the GRCh37 human reference genome. Then, we performed TraFic-mem using Docker image mobilegenomes/trafic:multispecies with default options. Then, we converted the coordinates to GRCh38.

Merge the results

Even for the identical SV, there are often slight deviations in inferred breakpoint coordinates across the software. Therefore, when SVs called by different software share the two breakpoints in close proximity (<= 10 bp), we deemed them as the same SV. GenomonSV, manta, and GRIDSS on Illumina sequencing data mostly produced equivalent coordinates of breakpoints whereas SvABA (at least the version we used) seemed not to provide non-exact breakpoint positions especially when the breakpoints share microhomology. Therefore, for the comparison of breakpoint coordinates, we did not use the results of SvABA.

Daily, Jeff. 2016. “Parasail: SIMD C Library for Global, Semi-Global, and Local Pairwise Sequence Alignments.” *BMC Bioinformatics* 17 (February): 81.

Kuhn, Robert M., David Haussler, and W. James Kent. 2013. “The UCSC Genome Browser and Associated Tools.” *Briefings in Bioinformatics* 14 (2): 144–61.

Li, Heng. 2013. “Aligning Sequence Reads, Clone Sequences and Assembly Contigs with BWA-MEM.” *arXiv [q-bio.GN]*. arXiv. http://arxiv.org/abs/1303.3997.

Rang, Franka J., Wigard P. Kloosterman, and Jeroen de Ridder. 2018. “From Squiggle to Basepair: Computational Approaches for Improving Nanopore Sequencing Read Accuracy.” *Genome Biology* 19 (1): 90.

Vaser, Robert, Ivan Sović, Niranjan Nagarajan, and Mile Šikić. 2017. “Fast and Accurate de Novo Genome Assembly from Long Uncorrected Reads.” *Genome Research* 27 (5): 737–46.

Wang, Ting, Lucinda Antonacci-Fulton, Kerstin Howe, Heather A. Lawson, Julian K. Lucas, Adam M. Phillippy, Alice B. Popejoy, et al. 2022. “The Human Pangenome Project: A Global Resource to Map Genomic Diversity.” *Nature* 604 (7906): 437–46.

Genomic Region A

Consensus sequence

	-	G	C	A	T	G	T	T	T	C	T	G	C	T
-	0	0	0	0	0	0	0	0	0	0	0	0	0	0
G	0	1	0	0	0	1	0	0	0	0	0	1	0	0
C	0	0	2	0	0	0	0	0	0	1	0	0	2	0
A	0	0	0	3	1	0	0	0	0	0	0	0	0	0
G	0	1	0	1	1	2	0	0	0	0	0	1	0	0
T	0	0	0	0	2	0	3	1	1	0	1	0	0	1
T	0	0	0	0	1	0	1	4	2	0	1	0	0	1
A	0	0	0	1	0	0	0	2	2	0	0	0	0	0
G	0	1	0	0	0	1	0	0	0	0	0	1	0	0
T	0	0	0	0	1	0	2	1	1	0	1	0	0	1
C	0	0	1	0	0	0	0	0	0	2	0	0	1	0
T	0	0	0	0	1	0	1	1	1	0	3	1	0	2
A	0	0	0	1	0	0	0	0	0	0	1	1	0	0
C	0	0	1	0	0	0	0	0	0	1	0	0	2	0
G	0	1	0	0	0	1	0	0	0	0	0	1	0	0

Genomic Region B

	-	T	T	A	T	A	T	A	T	C	T	A	G	G
-	0	0	0	0	0	0	0	0	0	0	0	0	0	0
G	0	0	0	0	0	0	0	0	0	0	0	0	1	1
C	0	0	0	0	0	0	0	0	0	2	0	0	0	0
A	0	0	0	3	1	3	1	3	1	0	0	3	1	0
G	0	1	1	1	1	1	1	1	1	1	1	1	4	4
T	0	2	2	0	2	0	2	0	2	0	2	0	2	2
T	0	1	3	1	1	0	1	0	1	0	1	0	0	0
A	0	2	2	5	3	5	3	5	3	2	2	5	3	2
G	0	0	0	3	3	3	3	3	3	1	0	3	6	4
T	0	2	2	1	4	2	4	2	4	2	2	1	4	2
C	0	0	0	0	2	2	2	2	2	5	3	1	2	2
T	0	1	1	0	1	0	3	1	3	3	6	4	2	0
A	0	0	0	2	0	2	1	4	2	1	4	7	5	3
C	0	0	0	0	0	0	0	2	2	0	2	5	8	6
G	0	0	0	0	0	0	0	0	0	0	0	3	6	9

$$H^1_{i,j} = \max \begin{cases} H^1_{i-1,j-1} + s(a_i, b^1_j) \\ H^1_{i-1,j} - g \\ H^1_{i,j-1} - g \end{cases}$$

$$H^2_{i,j} = \max \begin{cases} H^2_{i-1,j-1} + s(a_i, b^2_j) \\ H^2_{i-1,j} - g \\ H^2_{i,j-1} - g \\ \max_{i'} \{\max_{j'} H^1_{i',j'} + s\left(a_i, b^2_j\right) - j(i,i')\} \end{cases}$$

Supplementary Figure 1: Smith-Waterman algorithm with one-time jump to determine the SV breakpoints. A schematic of a one-time jump Smith-Waterman algorithm used to determine the exact breakpoints of structural variations. The first part of the consensus sequence is aligned to the genomic region A and the latter part is aligned to the genomic region B. This is basically the same as the standard Smith-Waterman algorithm except that one-time jump is allowed from genomic region A to B during the procedure, and the position where the jump occurred is determined to be the inferred breakpoint. When there are several inserted nucleotides, several bases of consensus sequences are also skipped during the jump.

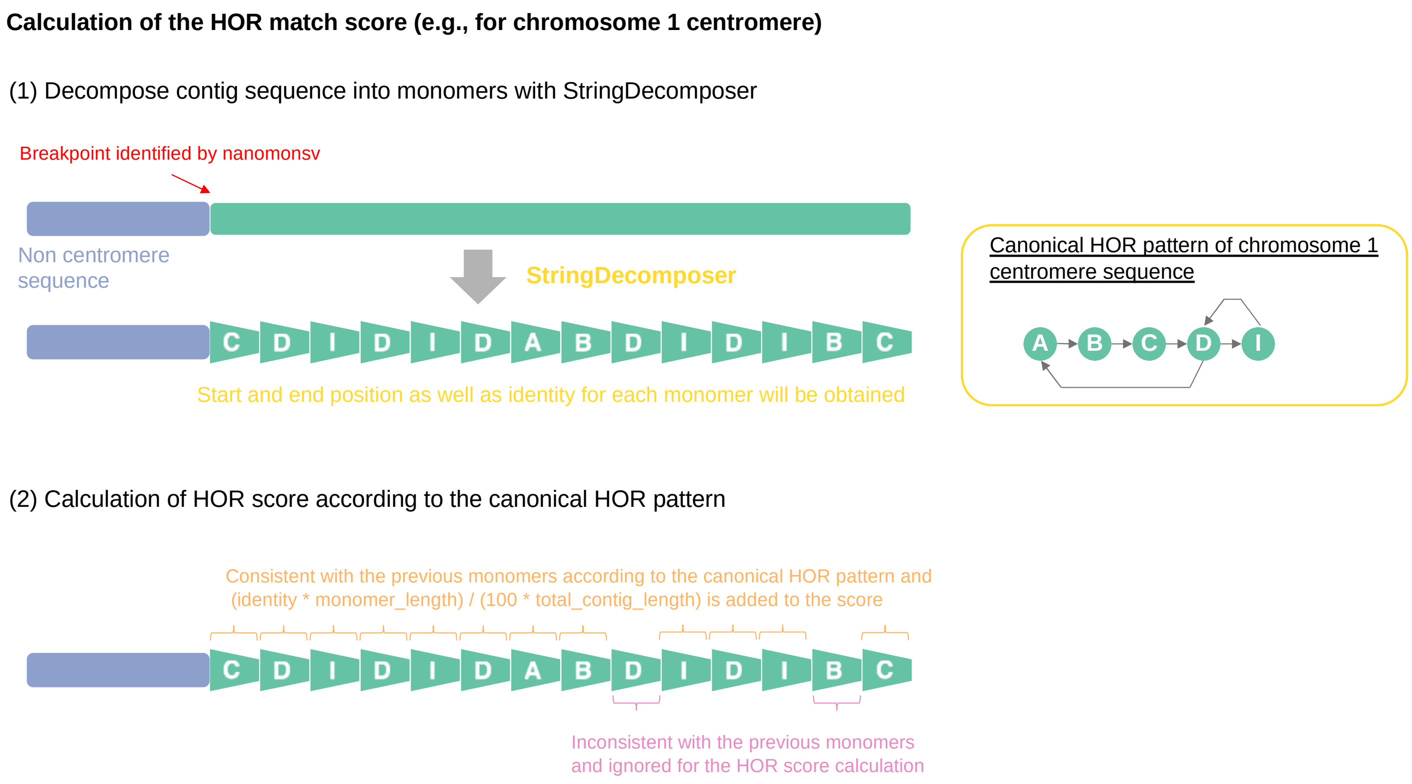

Supplementary Figure 2: Schematic of the HOR match score calculation. First, we decompose the contig sequence into monomers using StringDecomposer. Then, for each monomer, we check for consistency with the canonical HOR pattern represented by graphs and confirm that it is a child edge of the previous monomer. For the consistent monomers, we add the identity multiplied by the monomer length to the score. Finally, it is normalized by the total contig length.

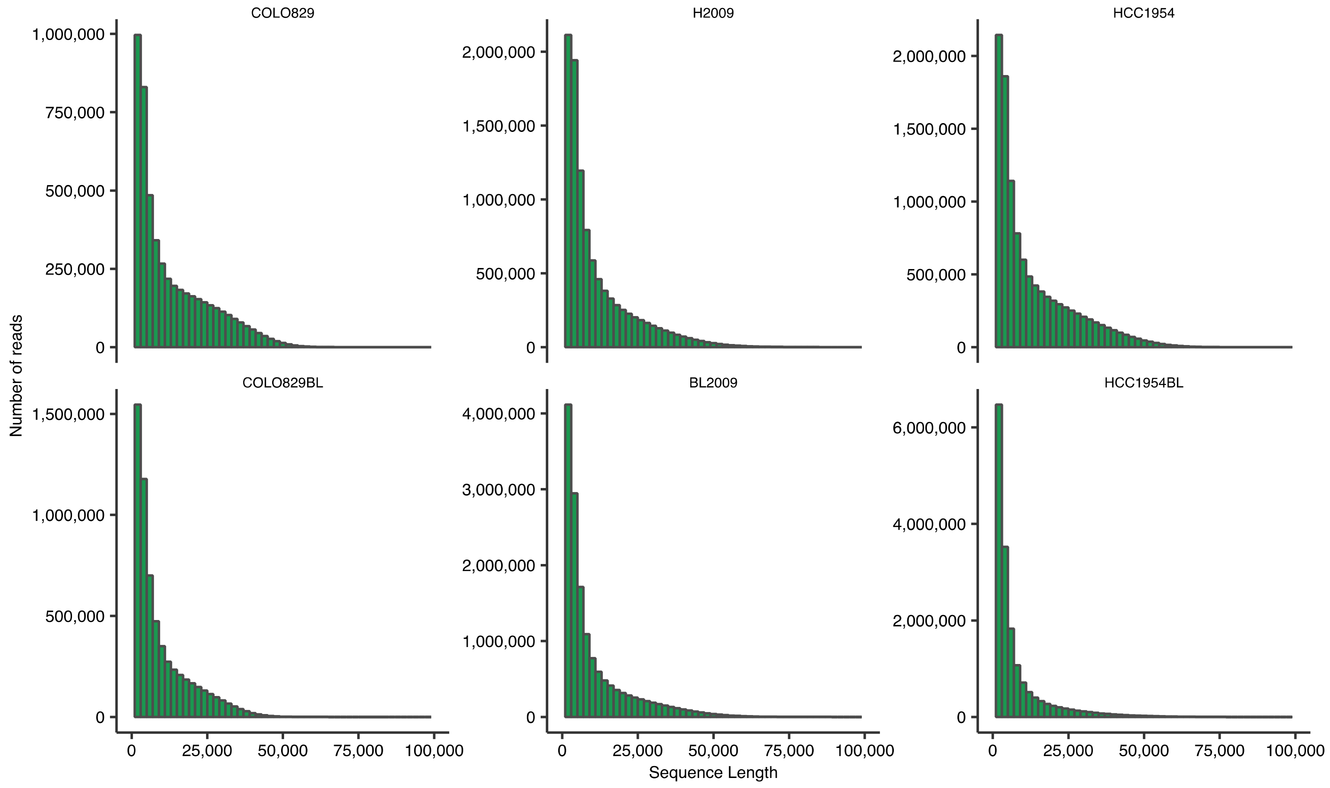

Supplementary Figure 3: Distribution of Nanopore read length for samples used in this study. Only primary alignment reads (those without either the secondary alignment (0x100) or the supplementary alignment (0x800) sam flag bits) were counted. The bin widths of the histograms are 2,000bp.

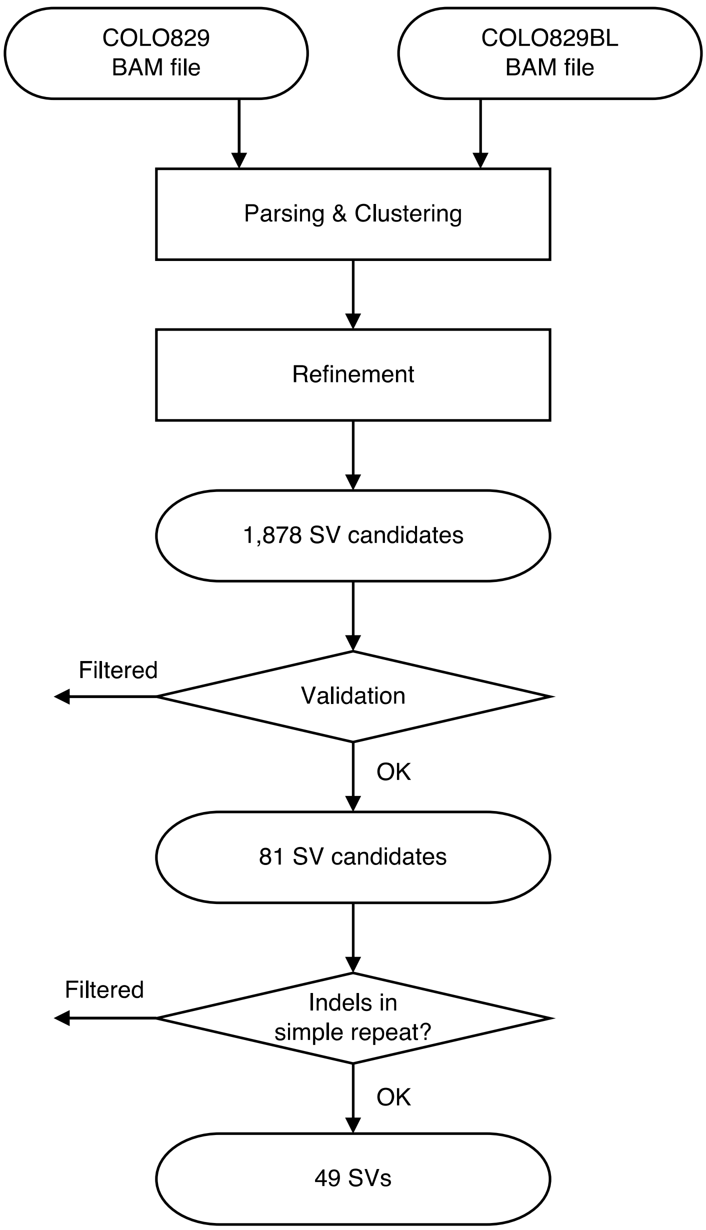

Supplementary Figure 4: Flowchart diagram of how many SV candidates remain in each module for COLO829. 1,878 SV candidates remained after the refinement step. Then, most SVs except 81 SV candidates were removed after the validation step. Finally, 49 SVs were called after removing indels confined to simple repeat regions.

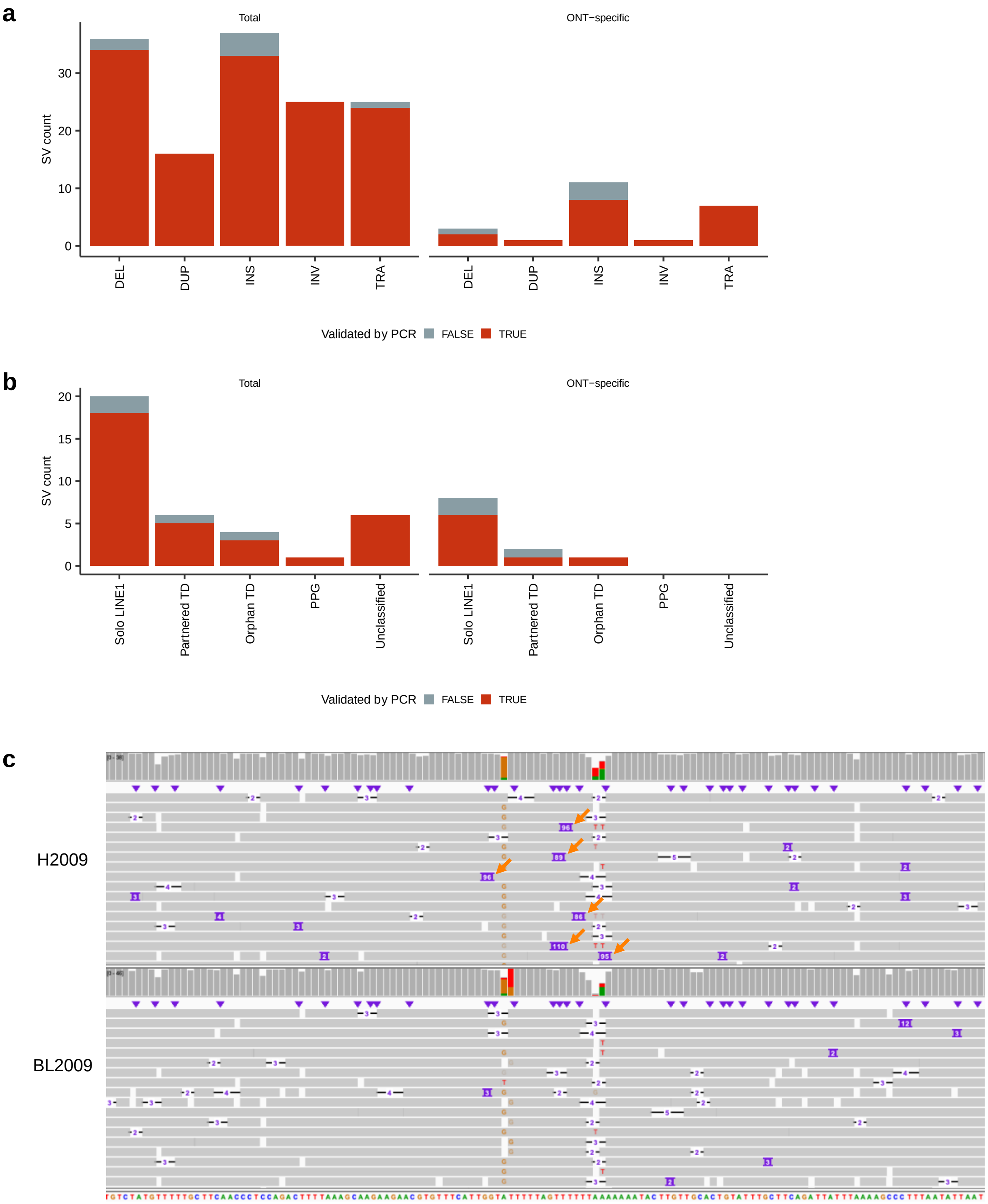

Supplementary Figure 5: Summary of PCR validation. (a, b) Numbers of SVs and insertions validated and not validated by PCR stratified by SV classes (DEL: deletion, DUP: duplication, INS: insertion, INV: inversion, TRA: translocation) and insertions, respectively. Partnered TD, Orphan TD, and PPG are partnered transduction, orphan transduction, and processed pseudogene, respectively. In the right panel, the result confined to SVs identified specifically by the long-read platform was shown. (c) An alignment view of one insertion (100 bp Solo LINE1 inserted at chr7:67665893). Even though this insertion could not be validated by PCR, supporting reads of the insertion (indicated by orange arrows) were observed in the tumor sample while no supporting reads were seen in the matched control.

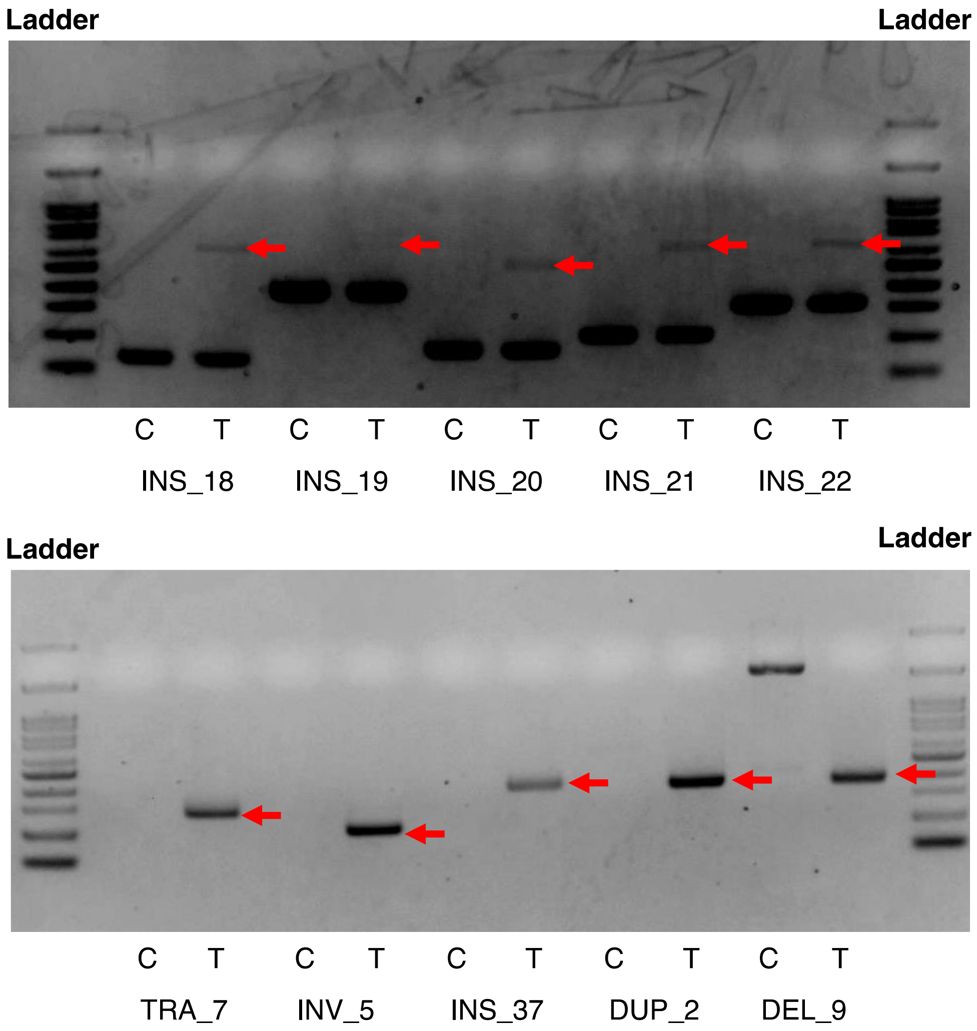

Supplementary Figure 6: Some somatic SVs validated by PCR for H2009. PCRs were performed on the tumor (T) and matched control (C) DNAs. The bottom keys correspond to the SV_ID in Supplementary Data 2. Bands for the target SVs were pointed by red arrows. For insertions, tumor-specific bands as well as common bands for tumor and control DNAs, which are shorter because of the lack of inserted sequences, were observed. For INS_34, the primer sequences are set on the genomic sequence around the insertion and the inserted sequence itself.

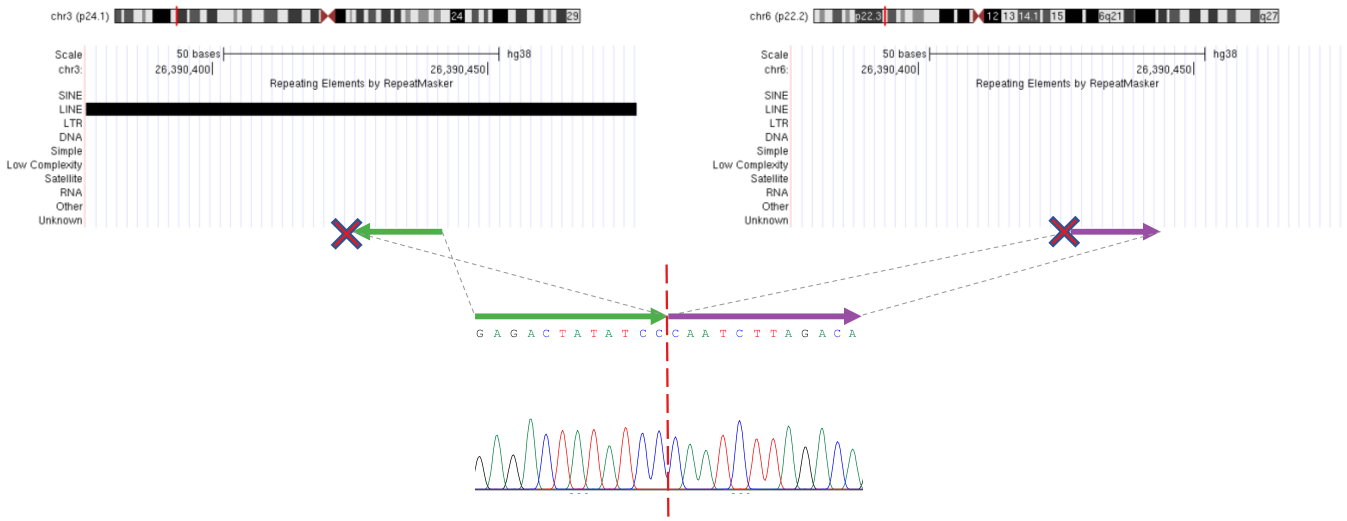

Supplementary Figure 7: Examples of somatic SVs identified specifically by long-read and its validation by Sanger sequencing. The somatic translocation, chr3:26,390,428 - chr6:26,193,811, in COLO829. The breakpoint at chromosome 3 is located in LINE1 sequences.

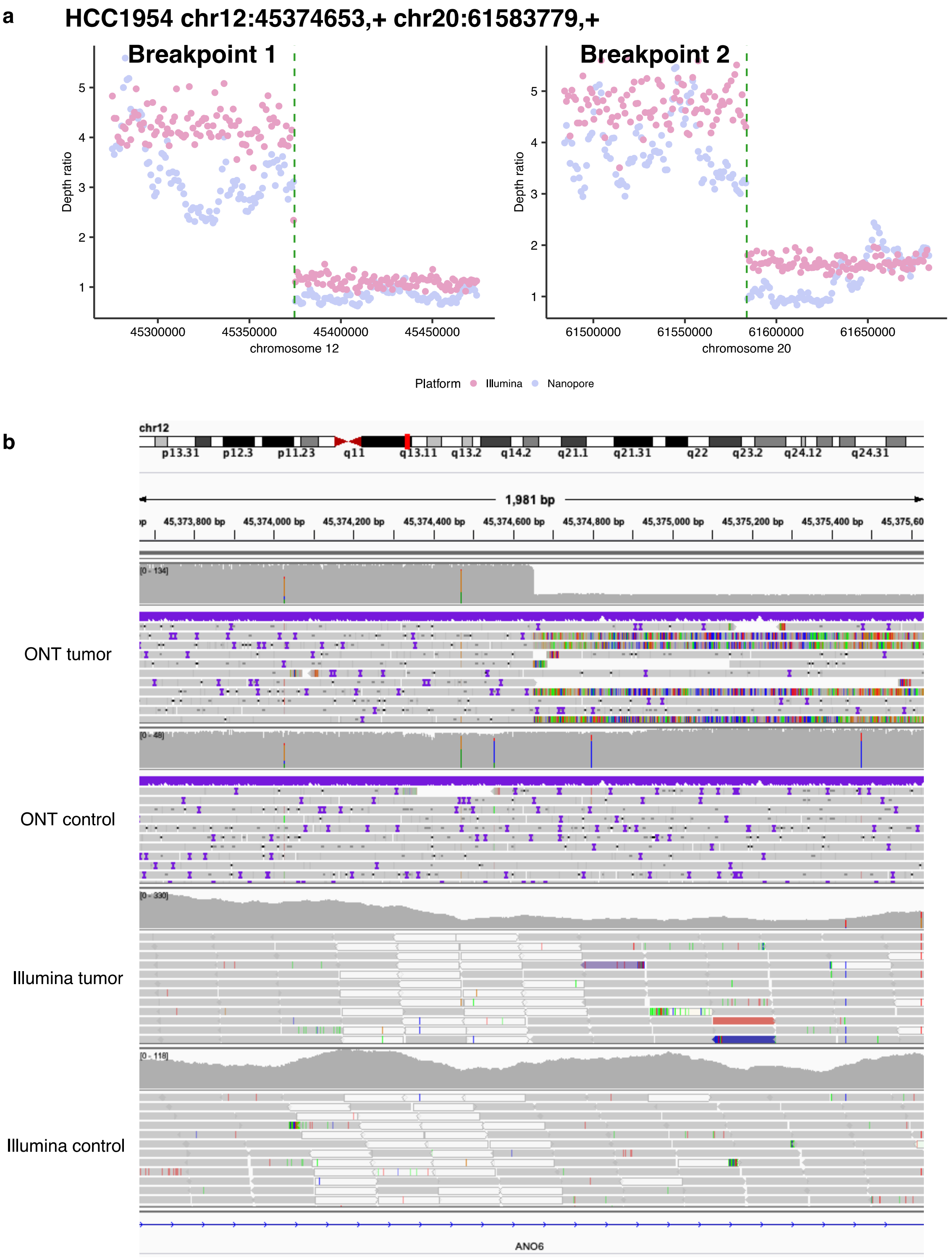

Supplementary Figure 8: Example of long-read specific SVs around with strong signal of copy number changes from HCC1945 (chr12:45,374,653 - chr20:61,583,779). (a) For each ONT and Illumina sequencing data, the ratios of sequence depth between tumor and matched control are calculated with the bin size of 1,000 bp and plotted for the surrounding 100,000 bp regions around each breakpoint. (b) The alignment figure via Integrative Genomics Viewer for the first breakpoint. The ambiguous alignment can be observed for Illumina sequence data (and that's why the short-read platform could not identify this SV), probably because the breakpoint is located in a LINE1 element.

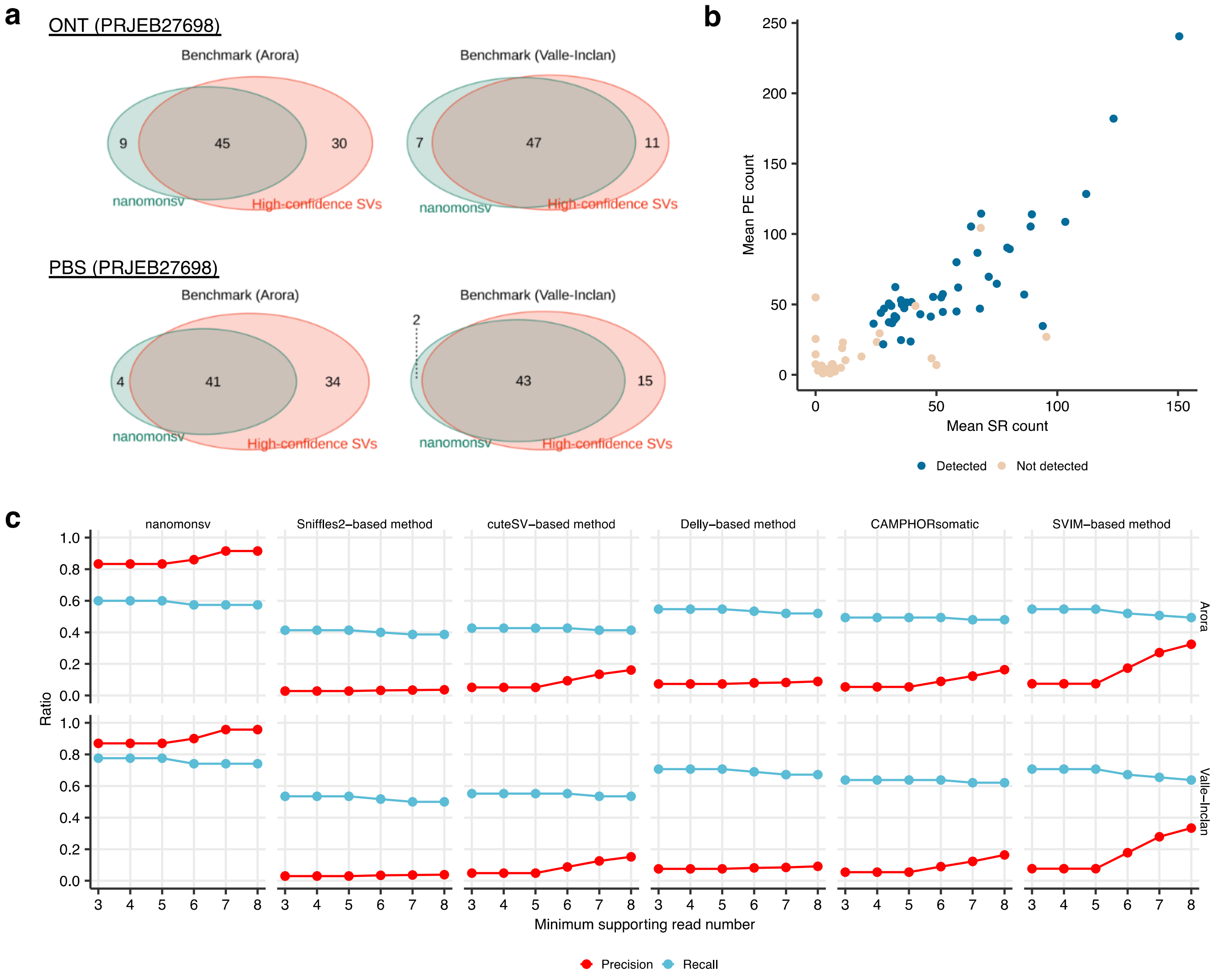

Supplementary Figure 9: Performance of nanomonsv on COLO829 benchmark dataset. (a) Overlap between SVs detected by nanomonsv and high-confidence SVs in COLO829 determined by two benchmark datasets (Arora et al. 2019[30] and Valle-Inclan et al. 2020[31]) on the different paired COLO829 sequencing data (Oxford Nanopore Technologies (ONT) and Pacific Biosciences (PBS) sequence data from PRJEB27698[31]) from our ONT data. See also Figure 3d. (b) Supporting reads of somatic SVs (mean split-read (SR) and paired-end (PE) read counts) presented in Arora et al. benchmark dataset[30] grouped by whether they are detected by nanomonsv or not. (c) The changes of precision and recall when changing the threshold of supporting read numbers by four different approaches on our COLO829 dataset.

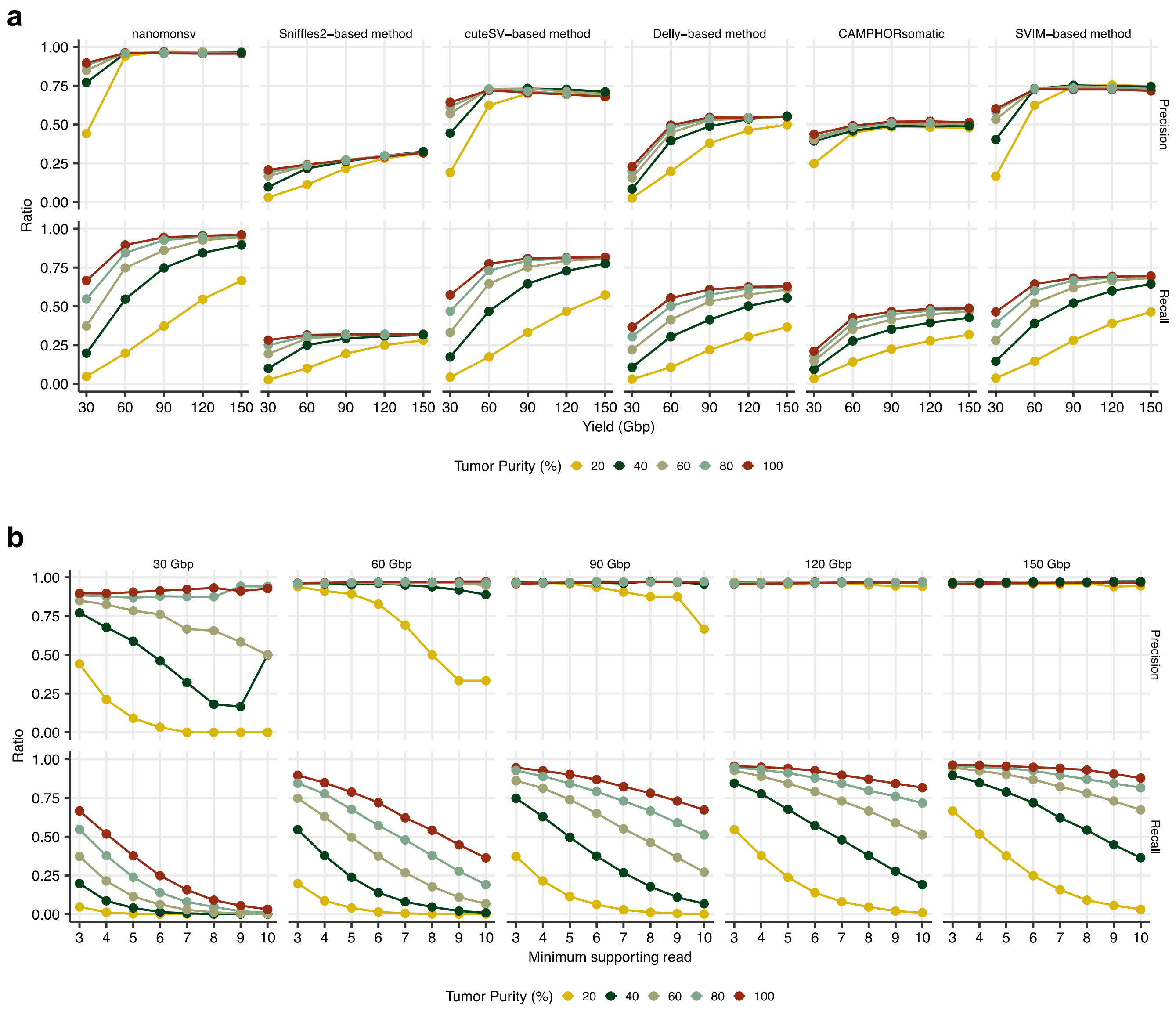

Supplementary Figure 10: Performance of nanomonsv and other approaches measured by simulation study. (a) Precision and recall of nanomonsv and five other SV detection approaches (Sniffles2, cuteSV, Delly, CAMPHORsomatic and SVIM) applied to simulated data with different tumor purities and sequence yields. For Sniffles2, cuteSV, Delly, and SVIM, separate detection and subtraction approaches were used, where regular SV detection tools were run separately on the tumor (>= 3 supporting reads) and matched control genomes (>= 1 supporting reads), and SVs detected from tumors were subtracted from those from normals. (b) The changes in precision and recall of nanomonsv with different thresholds of supporting reads.

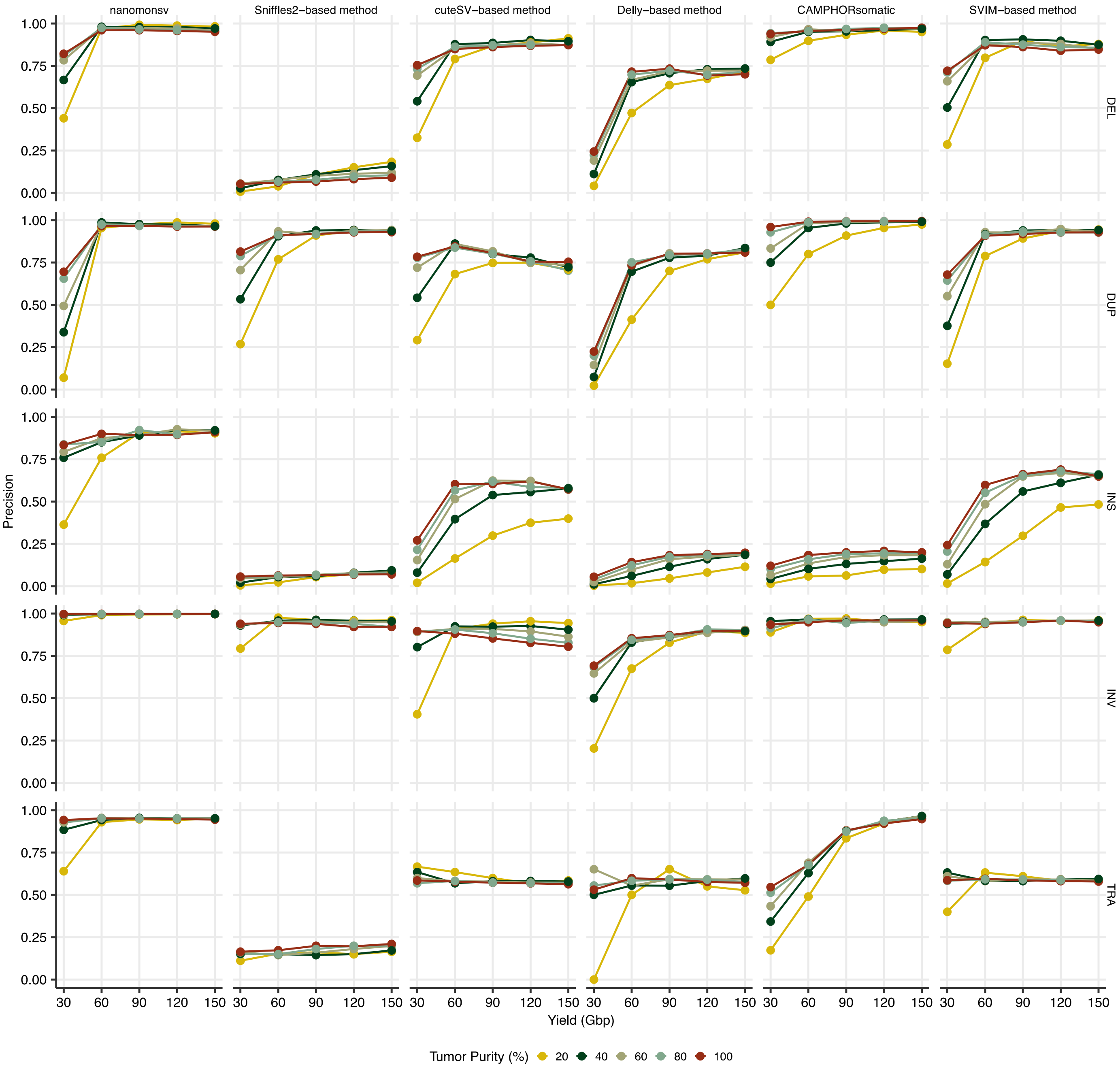

Supplementary Figure 11: Precision of nanomonsv and other approaches measured by simulation study stratified by SV classes. Precision of nanomonsv and five other SV detection approaches (Sniffles2, cuteSV, Delly, CAMPHORsomatic and SVIM) applied to simulated data with different tumor purities and sequence yields for each SV type (DEL: deletion, DUP: duplication, INS: insertion, INV: inversion, TRA: translocation). For Sniffles2, cuteSV, Delly, and SVIM, separate detection and subtraction approaches were used, where regular SV detection tools were run separately on the tumor (>= 3 supporting reads) and matched control genomes (>= 1 supporting reads), and SVs detected from tumors were subtracted from those from normals.

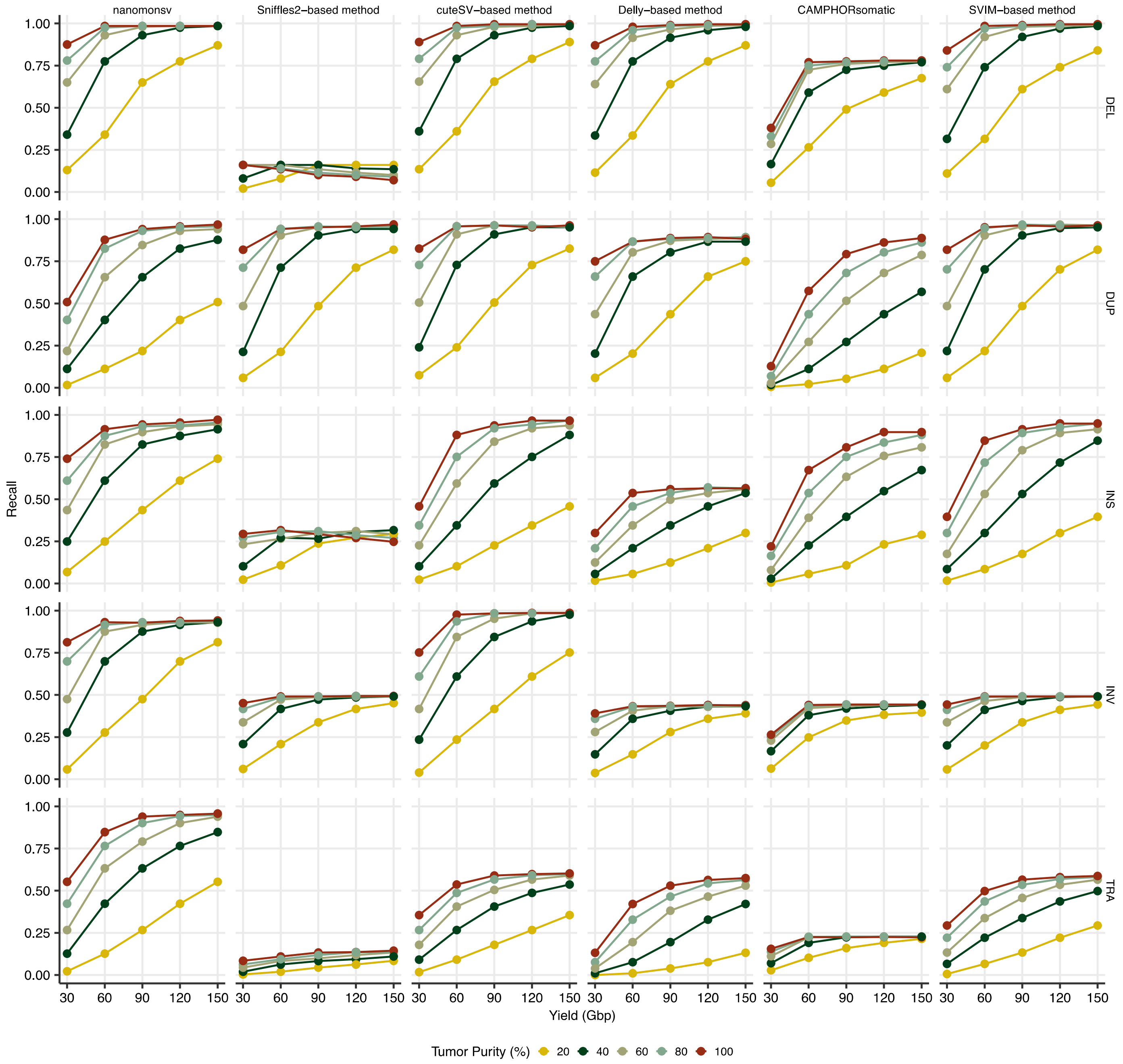

Supplementary Figure 12: Recall of nanomonsv and other approaches measured by simulation study stratified by SV classes. Recall of nanomonsv and five other SV detection approaches (Sniffles2, cuteSV, Delly, CAMPHORsomatic and SVIM) applied to simulated data with different tumor purities and sequence yields for each SV type (DEL: deletion, DUP: duplication, INS: insertion, INV: inversion, TRA: translocation). For Sniffles2, cuteSV, Delly, and SVIM, separate detection and subtraction approaches were used, where regular SV detection tools were run separately on the tumor (>= 3 supporting reads) and matched control genomes (>= 1 supporting reads), and SVs detected from tumors were subtracted from those from normals.

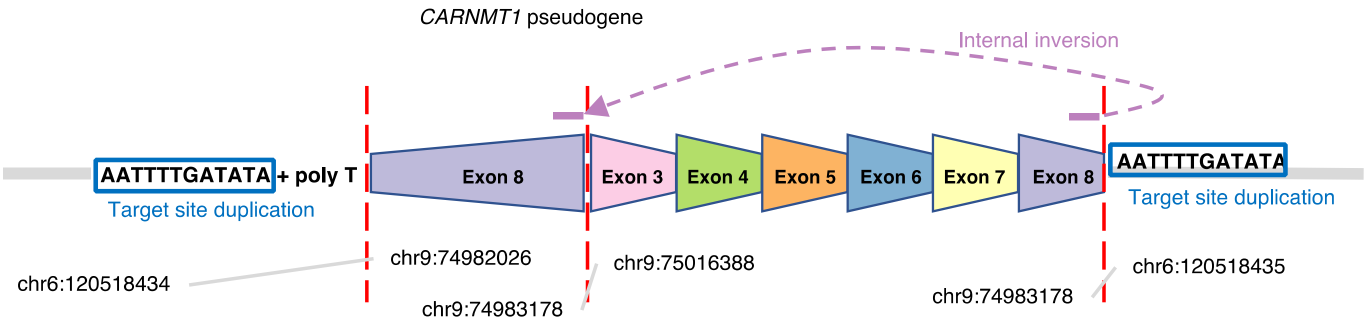

Supplementary Figure 13: Example of processed pseudogene insertion identified in H2009. A CARNMT1 pseudogene was somatically inserted into chromosome 6. Features commonly seen in LINE1 retrotransposition, such as a target site duplication, an internal inversion, polyA tail, and 5′ truncation were observed.

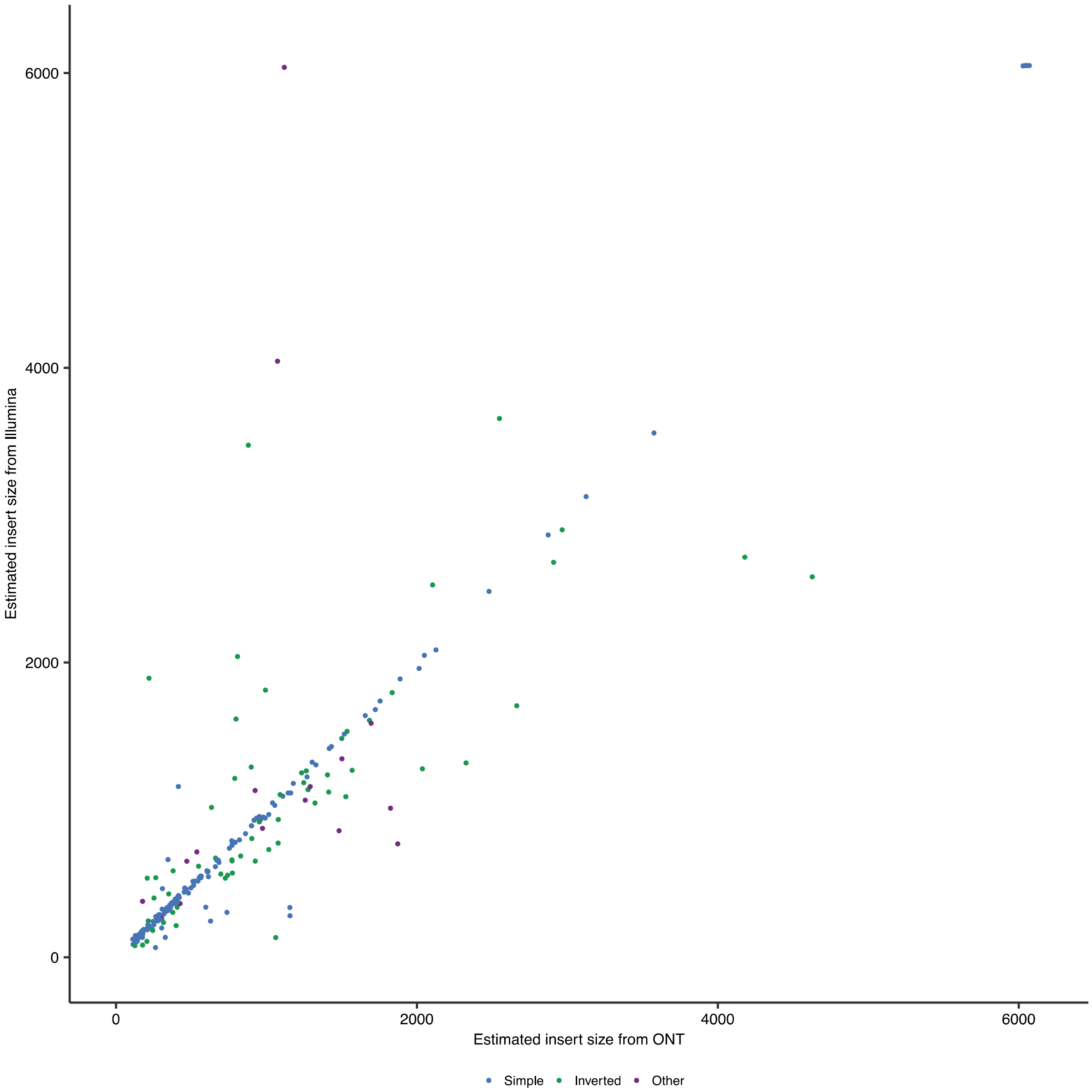

Supplementary Figure 14: Comparison between the estimated sizes of solo-L1 inserted sequences from Illumina and Oxford Nanopore Technologies data. Each point represents an insertion detected by both TraFic-mem on Illumina and nanomonsv on ONT sequence data. Insertions are stratified by the presence of an inversion, “Simple” (no inversions), “Inverted” (with one 5′ inversion), and “Other” (with multiple inversions). Complex LINE1 insertions (Inverted and Other) tend to have different insert size estimates.

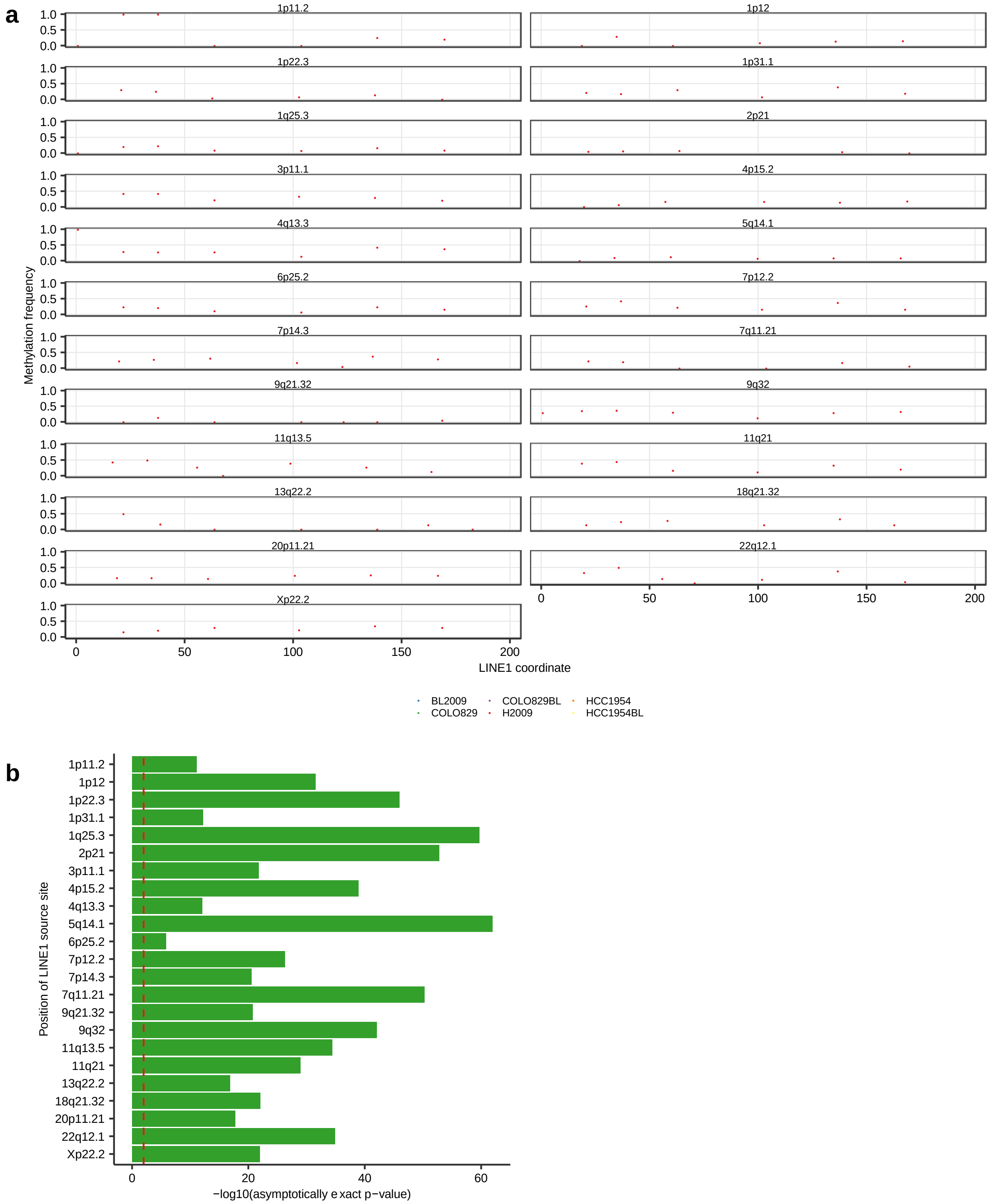

Supplementary Figure 15: The amounts of methylation of promoters of LINE1 source elements for H2009. (a) Methylation frequency at each CpG locus of each LINE1 source element for the tumor and the matched control represented by point plottings. When the CpG site by nanopolish was given as a region rather than a single point, the center point in the region was adopted as a coordinate. (b) P-values of the difference in methylation frequencies between the tumor and the matched control for each LINE1 source element. The vertical red dashed line indicates a significance level of 0.01.

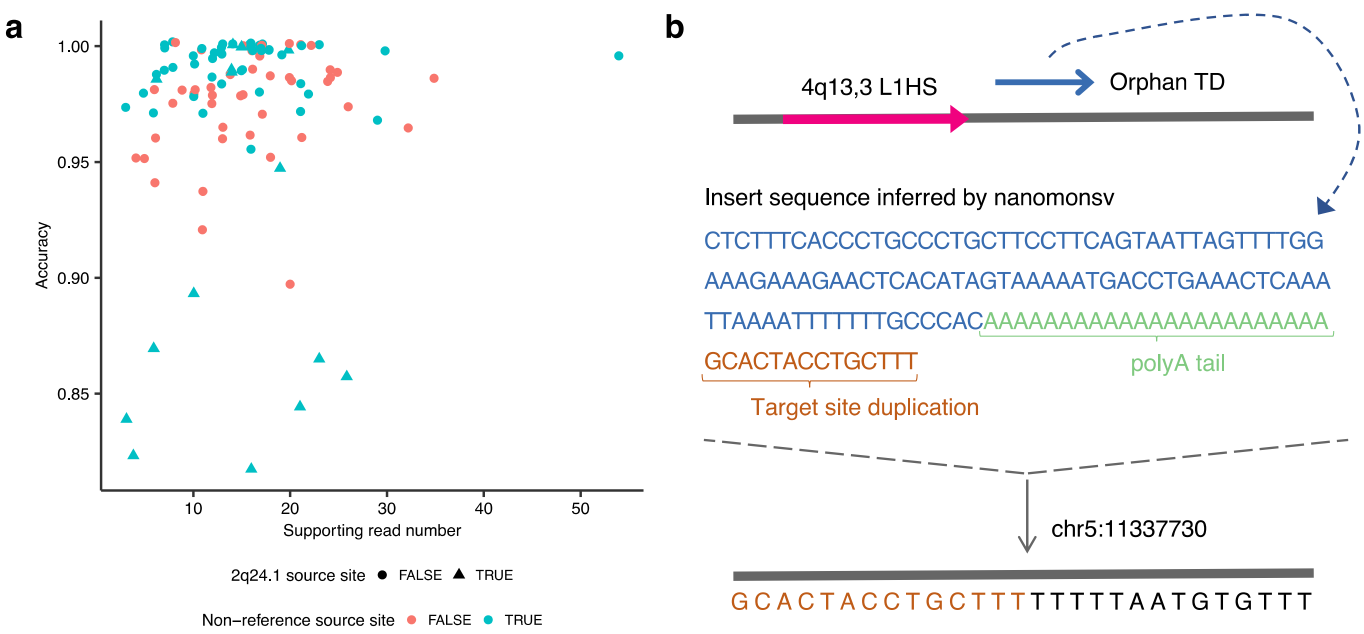

Supplementary Figure 16: Characterization of target site duplications and polyA tails. (a) Relationships between the accuracy of inserted sequences generated by nanomonsv and the number of supporting reads. Accuracy was estimated by the comparison with LINE1 transduction insertions and the reference genome sequences. In general, the accuracy increased with the number of supporting reads. Among eight inaccurate (<90%) sequences, seven out of the eight share the same non-reference source site at (2q24.1). Therefore, the inaccuracy may be due to some systematic mechanisms such as alignment errors around the source sites. (b) An example of an inserted sequence with target site duplications and polyA tails. This insertion on chromosome 5 is an orphan LINE1 transduction from the source site at 4q13.3. The genomic sequence from the downstream of the LINE1 source site is followed by polyA tails and duplicated parts of the target site.

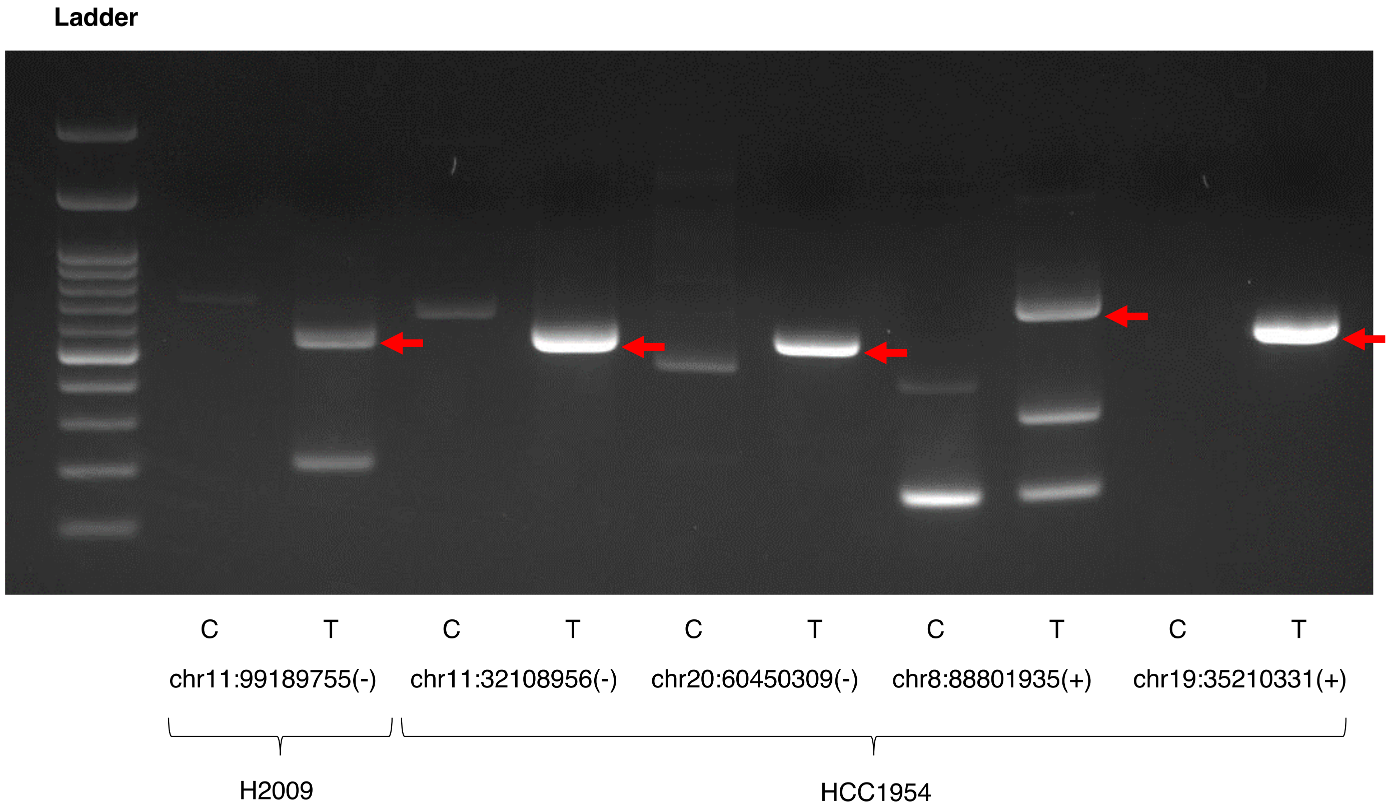

Supplementary Figure 17: Some somatic single breakend SVs leading to centromere sequences validated by PCR. PCRs were performed on the tumor (T) and matched control (C) DNAs. Bands for the target SVs were pointed by red arrows. See also Supplementary Data 5.

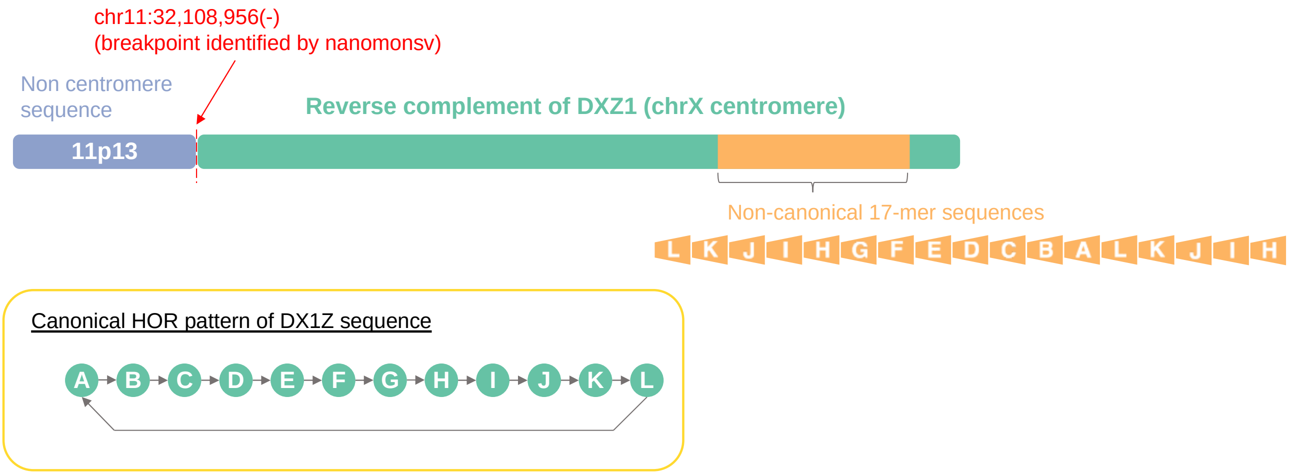

Supplementary Figure 18: A characteristic example of single breakend SV connected to alpha satellite sequence. This SV connects to the alpha satellite sequence (DXZ1) of the X chromosome, where usually 12 different monomer sequences (set as A ~ L) appear in sequence. Such a canonical pattern was observed in the vicinity of the breakpoint, albeit in the reverse complement. However, the 17-mer ABCDEFGHIJKLHIJKL sequence occurs twice in the middle. See also Figure 7c.

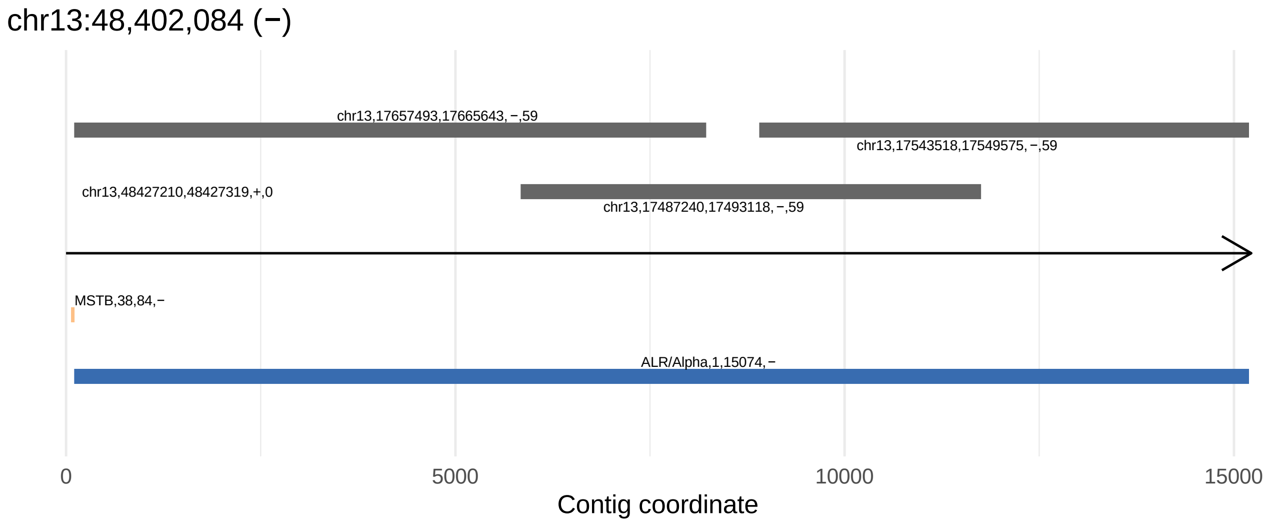

Supplementary Figure 19. The annotation result for the contig sequence of complex SVs involving centromere sequence affecting *RB1* gene in H2009. The upper part shows the alignment results to the human genome reference sequence (ambiguously matches the centromeric region of chromosome 13), and the lower part shows the repeat masker results (alpha satellite sequences). See also Figure 7b.

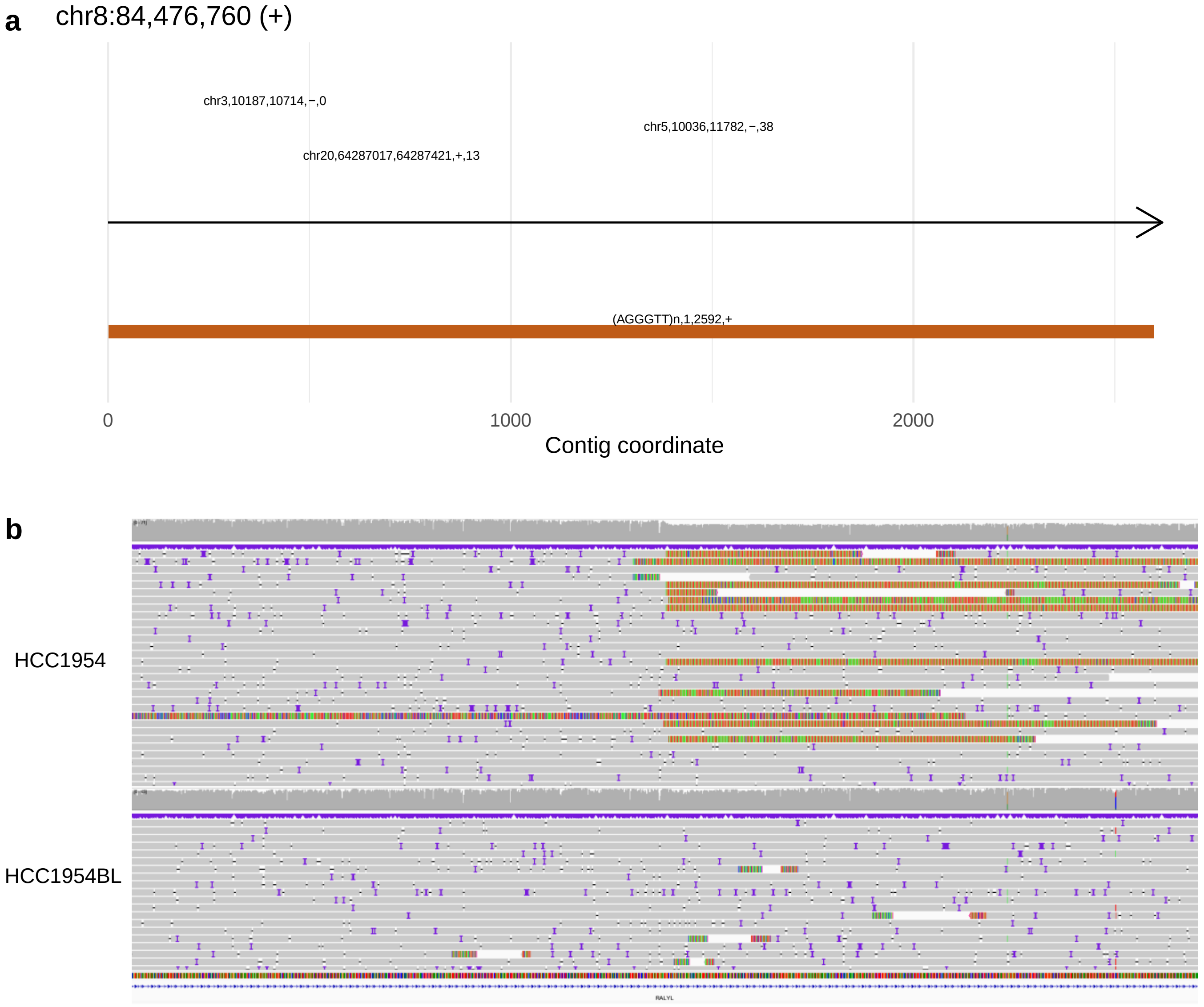

Supplementary Figure 20: Depiction of putative telomere insertion in HCC1954. (a) An annotation result for the contig sequence corresponding to a putative telomere insertion. The upper part shows the alignment results to the human genome reference sequence (ambiguously match the region near telomeres) and the lower part shows the repeat masker results [simple tandem repeat AGGGTT, which is a displaced frame of the canonical telomere element (TTAGGG)]. (b) The alignment figure via Integrative Genomics Viewer for the breakpoint. The soft-clipping parts consist of TTAGGG repeat arrays.

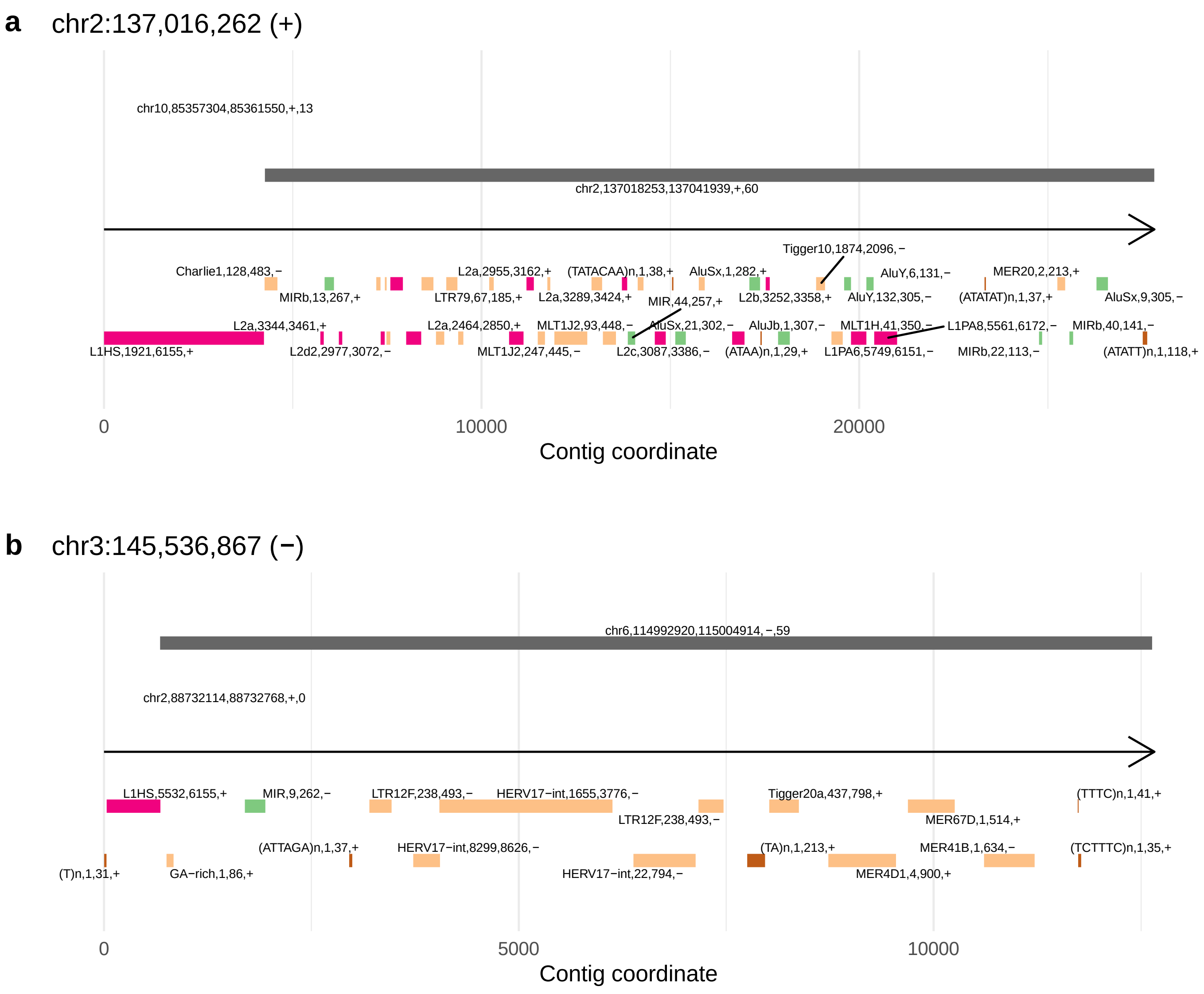

Supplementary Figure 21: Examples of annotation results for contig sequences corresponding to L1-mediated deletion and rearrangement. In each panel (a, b), the upper part shows the alignment results to the human genome reference sequence and the lower part shows the repeat masker results. Typically, the segments from near the breakpoint are annotated as L1HS (LINE-1 element L1 Homo sapiens) and their alignments to the human genome reference are ambiguous.

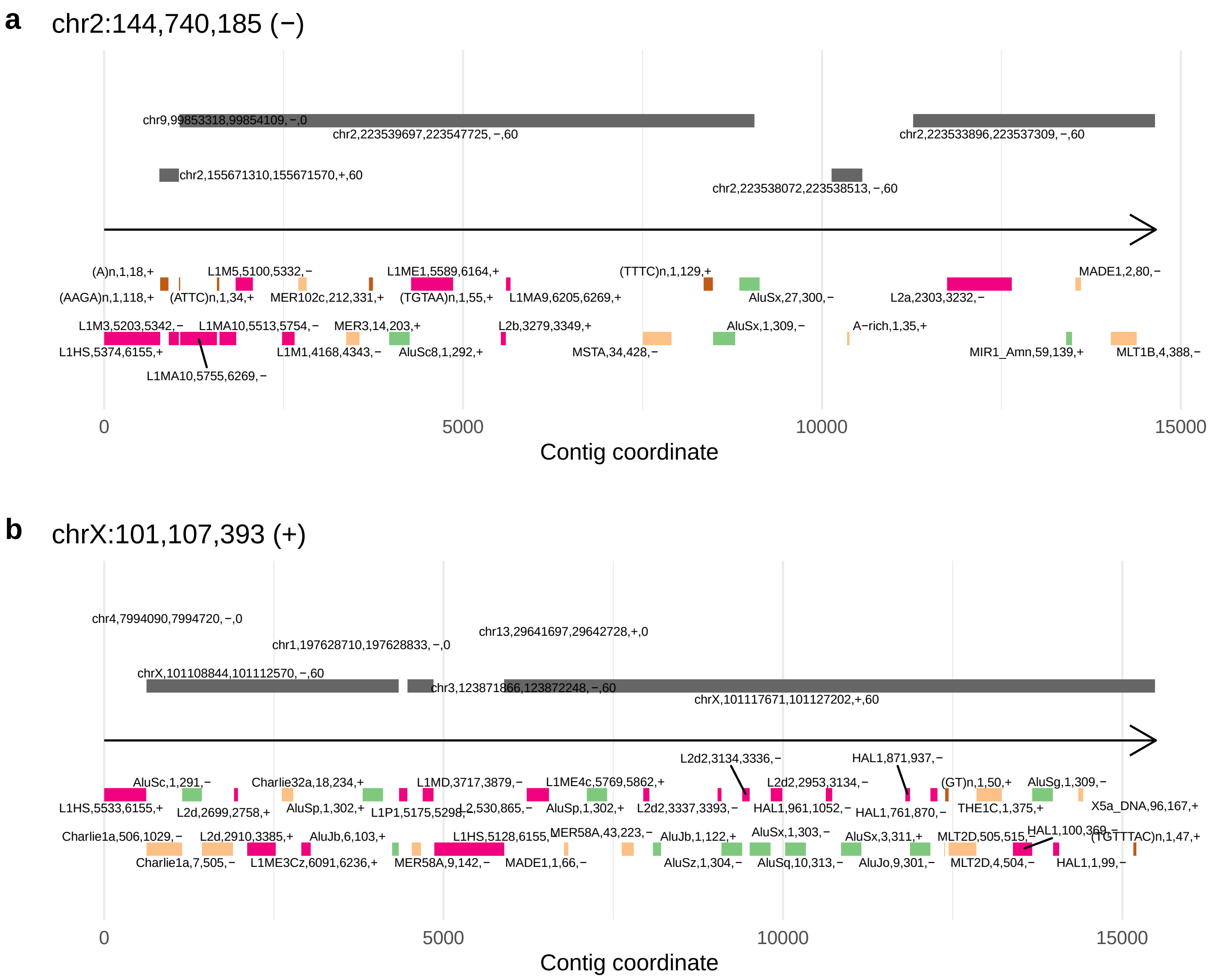

Supplementary Figure 22: Annotation results for contig sequences corresponding to complex SVs with LINE1-mediated rearrangements. In each panel (a, b), the upper part shows the alignment results to the human genome reference sequence and the lower part shows the repeat masker results. See also Figure 8a.

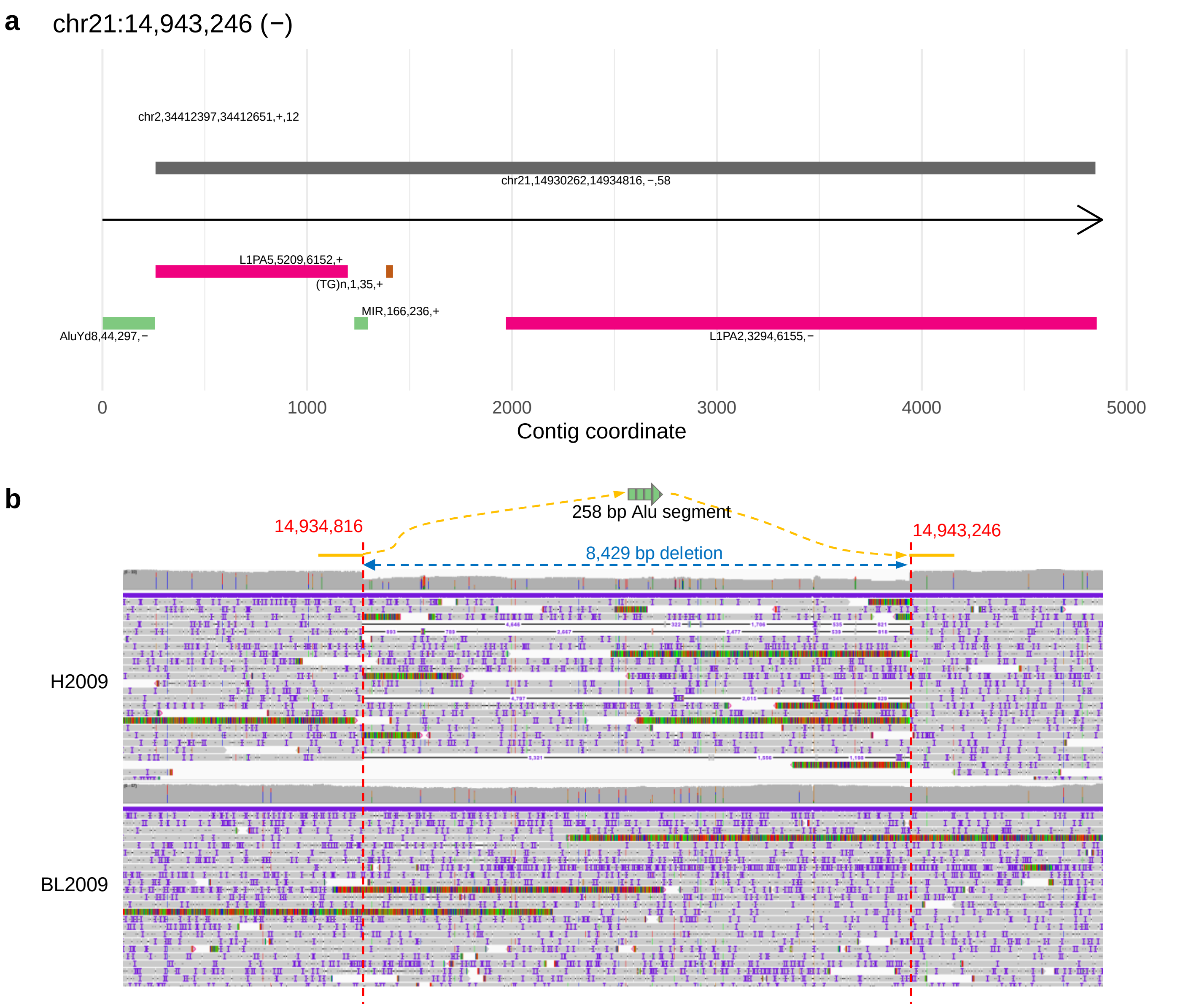

Supplementary Figure 23: Depiction of putative Alu-mediated deletion. (a) An annotation result for the contig sequence corresponding to the putative Alu-mediated deletion. The upper part shows the alignment results to the human genome reference sequence, where the first segment was ambiguously aligned and the second segment matched to the region near the breakpoint of the single breakend SV. (b) The alignment figure via Integrative Genomics Viewer for the breakpoint. The soft-clipping parts near the breakpoint matched to Alu sequence.
